# Partial Activation of SA- and JA-Defensive Pathways in Strawberry upon *Colletotrichum acutatum* Interaction

**DOI:** 10.3389/fpls.2016.01036

**Published:** 2016-07-15

**Authors:** Francisco Amil-Ruiz, José Garrido-Gala, José Gadea, Rosario Blanco-Portales, Antonio Muñoz-Mérida, Oswaldo Trelles, Berta de los Santos, Francisco T. Arroyo, Ana Aguado-Puig, Fernando Romero, José-Ángel Mercado, Fernando Pliego-Alfaro, Juan Muñoz-Blanco, José L. Caballero

**Affiliations:** ^1^Departamento de Bioquímica y Biología Molecular e Instituto Andaluz de Biotecnología, Edificio Severo Ochoa (C6), Universidad de CórdobaCórdoba, Spain; ^2^Instituto de Biología Molecular y Celular de Plantas, Universidad Politécnica de Valencia–Consejo Superior de Investigaciones CientíficasValencia, Spain; ^3^Departamento de Arquitectura de Computación, Universidad de Málaga, Campus de TeatinosMálaga, Spain; ^4^Centro Andalusian Institute of Agricultural and Fishering Research and Training (IFAPA) Las Torres-Tomejil, CAPMA–Junta de AndalucíaSevilla, Spain; ^5^Departamento de Biología Vegetal, Facultad de Ciencias, Universidad de Málaga, Campus de TeatinosMálaga, Spain

**Keywords:** *Colletotrichum acutatum*, *Fragaria* × *ananassa*, quantification of gene expression, salicylic and jasmonic acid, strawberry defense response

## Abstract

Understanding the nature of pathogen host interaction may help improve strawberry (*Fragaria* × *ananassa*) cultivars. Plant resistance to pathogenic agents usually operates through a complex network of defense mechanisms mediated by a diverse array of signaling molecules. In strawberry, resistance to a variety of pathogens has been reported to be mostly polygenic and quantitatively inherited, making it difficult to associate molecular markers with disease resistance genes. *Colletotrichum acutatum* spp. is a major strawberry pathogen, and completely resistant cultivars have not been reported. Moreover, strawberry defense network components and mechanisms remain largely unknown and poorly understood. Assessment of the strawberry response to *C. acutatum* included a global transcript analysis, and acidic hormones SA and JA measurements were analyzed after challenge with the pathogen. Induction of transcripts corresponding to the SA and JA signaling pathways and key genes controlling major steps within these defense pathways was detected. Accordingly, SA and JA accumulated in strawberry after infection. Contrastingly, induction of several important SA, JA, and oxidative stress-responsive defense genes, including *FaPR1-1, FaLOX2, FaJAR1, FaPDF1*, and *FaGST1*, was not detected, which suggests that specific branches in these defense pathways (those leading to *FaPR1-2, FaPR2-1, FaPR2-2, FaAOS, FaPR5*, and *FaPR10)* were activated. Our results reveal that specific aspects in SA and JA dependent signaling pathways are activated in strawberry upon interaction with *C. acutatum*. Certain described defense-associated transcripts related to these two known signaling pathways do not increase in abundance following infection. This finding suggests new insight into a specific putative molecular strategy for defense against this pathogen.

## Introduction

Strawberry fruit (*Fragaria* × *ananassa* Duch.) is of great importance throughout the world due to its flavor and nutritious qualities [FAOSTAT (http://faostat.fao.org/)] (Freeman et al., 2001). However, strawberry exhibits wide diversity in its susceptibility to a large variety of phytopathogenic organisms, including *Colletotrichum* spp., which are major pathogens of this crop (Simpson, 1991; Maas, 1998), requiring excessive use of chemical agents for disease control. Breeding for resistance by crossing in natural resistance mechanisms found in related genotypes lead to more sustainable farming with fewer chemical inputs. However, resistance to a variety of pathogens is generally polygenic and quantitatively inherited (Amil-Ruiz et al., 2011), making it difficult to associate single molecular markers with disease resistance genes.

Understanding the molecular interplay between plant and microbes has led to identification of candidate genes that have been used in developing transgenic strategies and breeding efforts to increase resistance against specific pathogens in many plants (Tohidfar and Khosravi, 2015). Plant resistance to pathogenic agents usually operates through a complex defense mechanism network. Compounds such as salicylic acid (SA), jasmonic acid (JA), and ethylene (ET) regulate plant defense pathways to trigger appropriate responses to different pathogens (Robert-Seilaniantz et al., 2011). Whereas the SA signaling pathway is mainly activated against biotrophic pathogens, the JA/ET signaling pathway is activated against necrotrophic pathogens. Interplay and antagonism between these signaling pathways has also been described (Robert-Seilaniantz et al., 2011). Pathogens have evolved to overcome plant immunity mechanisms by disrupting the fine crosstalk between these defense pathways (El Oirdi et al., 2011; Rahman et al., 2012; Chung et al., 2013).

In Arabidopsis, genetic markers have been identified to denote activation of canonical SA- and JA defense pathways. Both *PAD4* and *EDS1* activate SA biosynthesis, and both are also SA induced (Rustérucci et al., 2001; Venugopal et al., 2009). *PR1* and *PR2* are known pathogen- and SA-responsive genes, which are well-established markers for the *Arabidopsis* defense responses against *P. syringae* (Uknes et al., 1992). *WRKY70* encodes a transcription factor and is an important regulator in the interplay of SA- and JA-related plant defense responses (Li et al., 2004). *AOS* encodes an allene oxide synthase, which is responsible for JA synthesis, and its expression is also regulated by this hormone (Turner et al., 2002). *PDF1.2* and *JAR1* are genes that are commonly used to monitor JA responses (Staswick and Tiryaki, 2004; Pieterse et al., 2009), whereas *LOX2*, which is involved in JA biosynthesis, is activated by a positive feedback loop (Sasaki et al., 2001). *GST1* is part of the array of defense-related genes induced in response to oxidative burst produced after pathogen infection (Bhattacharjee, 2012). This gene encodes a glutathione S-transferase that is known to play a key role in reactive oxygen species (ROS) detoxification and reduction.

In strawberry, isolation of individual genes related to plant defense has been previously reported (Amil-Ruiz et al., 2011). Furthermore, Casado-Díaz et al. (2006) first reported isolation of a large set of genes with altered expression during strawberry and *C. acutatum* interaction. Over the last decade, microarrays have proved to be a valuable tool to analyze the expression of thousands of genes on a simultaneous basis, contributing to elucidate the underlying networks of gene regulation that lead to a wide variety of defense responses. Thus, Maleck et al. (2000) have provided a comprehensive description of the SAR genes from *Arabidopsis thaliana*. Wang et al. (2005) reported that SAR requires protein secretory pathway induction. Further, microarrays have led to numerous findings of key regulatory genes for defense signaling as well as valuable end-point genes whose products display direct action against pest and diseases (Wang et al., 2006; Sarowar et al., 2011). In addition, microarray analysis has demonstrated a substantial crosstalk among different defense signaling pathways (Schenk et al., 2003) and ultimately among genes and their products, and the entire pathways are not always tuned by signaling (Lodha and Basak, 2012). In strawberry, microarrays have also been used to analyze gene expression differences between white and red fruit after 24 h interaction with *C. acutatum* and provision of certain data from factors associated with pathogen quiescence during fruit immature stages (Guidarelli et al., 2011). However, strawberry defense network components remain largely unknown or poorly understood, and accurate mechanisms remain elusive.

In this report, a transcriptomic approach has been used to identify pathogen-responsive genes in *F* × *ananassa* strawberry crown and petiole, key sites of *C. acutatum* infection. Transcript accumulation was also monitored in response to SA and JA treatments. Synthesis of SA and JA signaling molecules was also analyzed. These trials reveal changes in the transcriptomic profile of plants challenged with *C. acutatum* and present a hypothesis about a strategy potentially used by this pathogen to overcome strawberry defense. These data deepen our understanding of the complex genetic and molecular mechanisms of strawberry defense.

## Materials and methods

### Plant materials, pathogen inoculation, and hormonal treatments

Plant culture (*Fragaria* × *ananassa* cultivar Camarosa) and growth conditions, *C. acutatum* (isolate CECT 20240) inoculation, and treatments with chemicals have been previously described (Encinas-Villarejo et al., 2009). In breaf, 8-week-old strawberry plantlets were placed in 20 cm diameter plastic pots containing sterilized peat and grown for a minimum of 6 additional weeks prior to mock or pathogen inoculation by spraying a spore suspension of 10^4^ conidia·ml^−1^. Crown was collected 5 days after treatment (spray-infected and mock-treated) for microarray studies. At this time, under our experimental conditions, plants still looked healthy, and no visible disease symptoms were easily detected, even in petioles, crowns or leaves. For RTqPCR analysis, crowns and petioles were collected 1, 3, 5, 7, and 9 days after treatment, as previously described in Casado-Díaz et al. (2006). For treatment and hormonal content analysis, axenic *in-vitro* plantlets were used and aseptically sprayed with either MeJa (2 mM) or SA (5 mM) solutions, or inoculated with *C. acutatum* conidia suspension (10^4^ conidia·ml^−1^), respectively. All collected plants were immediately frozen in liquid nitrogen and stored at −80°C until use. For transmission electron microscopy, pathogen was spot inoculated in crown by applying 50-μL droplets of conidia suspension (10^6^ conidia·ml^−1^) (Arroyo et al., 2005). Control plants were similarly inoculated with 50 μL of sterile distilled water. The position of each infection site was marked for reference. Inoculated plants were enclosed in plastic bags for 48 h to maintain high relative humidity and incubated in similar conditions as described above. Hence, disease is forced to progress quicker than by spraying.

### Light microscopic fungal development observation

Light microscopy analyses of *C. acutatum* development were performed on strawberry leaf discs (10 mm diameter) randomly excised from infected leaflet at 1, 3, 5, 7, 9 dpi, using a method modified from Debode et al. (2009). In breaf, leaf discs were cleared in 0.15% trichloroacetic acid (TCA) in a 3:1 (v/v) mixture of ethanol and chloroform for 48 h, with at least three changes of the bleaching solution, rinsed briefly in lactoglycerol and incubated at room temperature for 1 h in lactophenol blue (Sigma), and washed 3 times in lactoglycerol. For ROS detection, leaf discs were infiltrated in a 10 mg/ml DAB (3,3′ -diaminobenzidine, Sigma) solution for 10 min and subsequently incubated overnight at room temperature in dark, and cleared for 24 h in 3:1 (v/v) ethanol: glacial acetic acid with three changes. Treated leaf disc was mounted in 50% fresh glycerol on glass slides and examined using a Leica DM5000B microscope. Images were captured with a Leica DC500 digital camera. Three leaftlets were sampled from each plant, and 3 plants were observed at each time point. The overall number of conidia (germinated and non-germinated) and appresoria were counted per leaf disc taken up to 9 dpi.

### Transmission electron microscopy (TEM)

Strips of tissue ~1 mm thick and 1–2 mm long were removed from beneath the inoculation droplets and fixed in 4% (v/v) glutaraldehyde in 0.1 mol·L –1cacodylate buffer (pH 7.2) for 3 h at 4°C. Upon rinsing in the same buffer, tissues were post-fixed in 1% (w/v) osmium tetroxide for 2 h at 4°C and subsequently dehydrated in a graded acetone series and embedded in EMBED-812 (Polysciences, Warrington, Penn.) according to manufacturer's instructions. Slides with semithin sections (0.5 μm) were placed on a hotplate at 50°C, stained for 1 min with 0.1% aqueous toluidine blue O and examined using a light microscope (Leitz Aristoplan). Ultrathin 60–80 nm sections were made with a Reichert-Jung Ultracut E ultramicrotome and a diamond knife, and collected on 300-mesh copper grids (Dashek and Mayfield 2000). Grids were stained with 7% aqueous uranyl acetate and lead citrate. Sections were observed and images were collected using a Philips CM-10 transmission electron microscope (TEM).

### Total RNA extraction and real-time qPCR

Total RNA from strawberry tissues was isolated as described previously (Casado-Díaz et al., 2006), treated with DnaseI (Invitrogen) for residual DNA removal, and further purified with the RNeasy MinElute Cleanup Kit (QIAGEN). Purified RNA was quantified by NanoDrop 1000 Spectrophotometer (Thermo scientific). RNA integrity was checked using the Agilent 2100 Bioanalyzer (Agilent Technologies, Deutschland). First-strand cDNA synthesis was carried out using 1 μg of purified total RNA as template for a 20 μL reaction [iScript cDNA Synthesis kit (Bio-Rad)]. RT reactions were diluted 5-fold with nuclease-free water prior to qPCR.

Specific primer pairs set were designed using Oligo Primer Analysis software version 6.65, tested by dissociation curve analysis, and verified for absence of non-specific amplification (Table S1). FaGAPDH2 gene was used for normalization (Khan and Shih, 2004; Amil-Ruiz et al., 2013). RTqPCR runs were performed using two technical replicates in the same run and three biological replicates in different runs, as described previously (Encinas-Villarejo et al., 2009), using SsoAdvanced™ SYBR® Green supermix, and MyIQ v1.004 and iCycler v3.1 real-time PCR systems (Bio-Rad).

### Microarray analysis and strawberry gene annotation

For microarray analysis, strawberry samples were collected 5 days after treatment (spray-infected and mock-treated). Crowns were used to make biological replicates, and overall RNA was isolated from three independent biological replicates for hybridization against a proprietary microarray representing approximately 2529 predicted unigenes from *F. vesca* (Shulaev et al., 2011), previously identified from strawberry libraries (Casado-Díaz et al., 2006; and JL Caballero unpublished). Microarray data with accession GSE56296 were deposited in the NCBI Gene Expression Omnibus. Quality control, labeling, hybridization, and scanning were carried out by the SCAI, University of Córdoba (http://www.uco.es/servicios/scai/index.html), following the Genomic Unit guidelines. Microarray images were analyzed using GenePix 6.0 software (Molecular Devices). Data were transformed using an intensity-based Lowess function (Yang et al., 2002) with Acuity 4.0 software (Axon Instruments). Genes were considered as differentially expressed if they fulfilled both a FDR < 0.05 after a SAM test analysis (Tusher et al., 2001), and the fold-change (up or down) was above 1.75-fold.

To assign a putative biological function to every detected differentially expressed gene, their respective orthologous genes from the wild species *F. vesca*, which genome has been recently released (Shulaev et al., 2011), were identified by blasting the EST sequence associated with each singular spot within the array to the overall collection of *F. vesca* predicted genes (Altschul et al., 1990; Shulaev et al., 2011; http://www.rosaceae.org/). In order to enrich this process, *A. thaliana* putative orthologs were also identified for every *F* × *ananassa* gene as vast functional information is available for the former species (TAIR10: http://www.arabidopsis.org/). FunCat and GO terms assignments were first used to perform an automated functional categorization of differentially expressed genes (Ashburner et al., 2000; Ruepp et al., 2004). FatiGO tool (a web tool to find significant associations of Gene Ontology terms with gene groups; Ashburner et al., 2000; Ruepp et al., 2004) was used to perform a comprehensive Singular Enrichment Analysis (SEA) to extract relevant GO terms associated with up-regulated genes. Briefly stated, it takes two lists of genes (ideally a group of interest and the remaining genes in the experiment, while any two groups, formed in any way, can be tested against each other) and converts them into two lists of GO annotations using the corresponding gene or protein—term annotation table. A Fisher's exact test for 2 × 2 contingency tables is subsequently used to check for significant over-representation of GO annotations in one of the sets with respect to the other. Multiple test correction to account for the multiple hypotheses tested (one for each functional term) is applied. The terms are considered to be relevant by the application of statistical tests, as described in Al-Shahrour et al. (2004).

### Hormone determination in strawberry tissues

Extraction and purification procedures and chromatographic analysis have been previously described (Durgbanshi et al., 2005). In short, 3 grams of frozen green tissue were lyophilized and immediately homogenized in 5 mL of ultrapure water. After centrifugation (5000 g, 10 min), supernatant pH was adjusted to 2.8 with 15% (v/v) CH3COOH, and the supernatant was partitioned twice against an equal volume of diethyl ether. The aqueous phase was discarded and the organic fraction was vacuum evaporated at room temperature. The solid residue was resuspended in 1 mL of a 90:10 (v/v) water/methanol solution and subsequently filtered through a cellulose acetate filter (0.22 μm). A 20 μL aliquot of this solution was then injected into the high performance liquid chromatography (HPLC) system from Waters, Milford MA (Alliance 2690 system). Aliquots were injected on a Nucleosil ODS reversed-phase column. Phytohormones were eluted with a gradient of methanol and 0.01% CH3COOH in water that started from 10:90 (v/v) and linearly reached 60:40 (v/v) in 10 min. In the following 4 min, the gradient was increased to 80:20 (v/v). Isocratic conditions of 80:20 (v/v) were then retained during the last 2 min of the run. Initial conditions were restored and allowed to equilibrate for 5 min, for a total time of 21 min per sample. The solvent flow rate was 0.3 mL/min, with working pressures at around 70–100 bar.

Quoted plant hormone endogenous contents are mean values from 2 measurements of each of all 3 biological replicates. The One-way Analysis of Variance (ANOVA) with a Bonferroni Multiple Comparisons Test was performed using GraphPad InStat3 for Windows (GraphPad Software, La Jolla California USA, www.graphpad.com) to calculate the significant differences between control and inoculated plants.

## Results

### Monitoring *C. acutatum* development in plant tissue

The histopathology of the interaction strawberry-*C. acutatum* has been previously reported (Curry et al., 2002), and the transition to the necrotrophic phase was established after 4 dpi (Horowitz et al., 2002). However, fungal colonization in plant may vary with infection conditions. Thus, we monitored the infection progress of *C. acutatum* on strawberry plant after artificial inoculation (spraying) using microscopic analysis.

**Figure 1:**
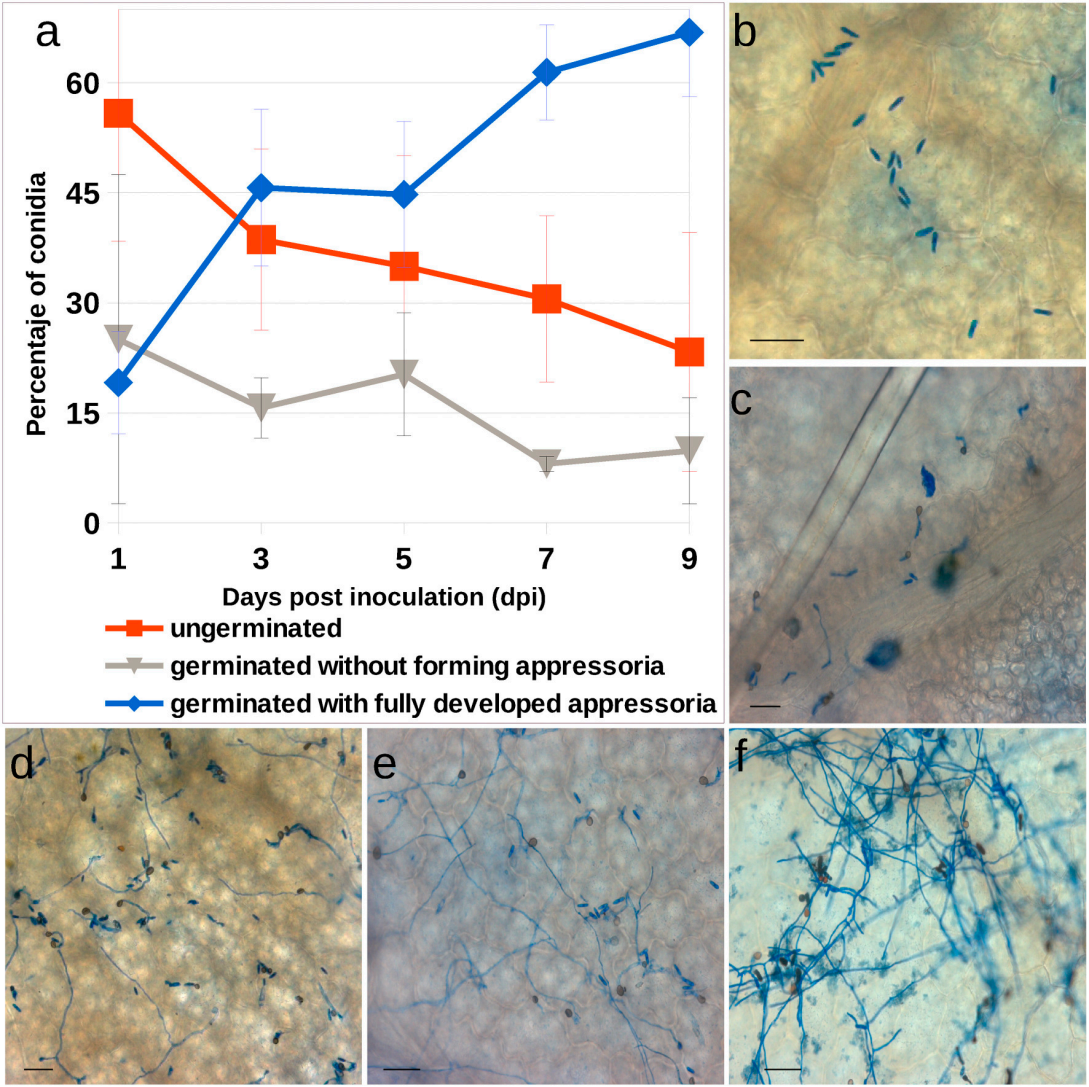
***Colletotrichum acutatum* development in strawberry tissue. (A)** Progression of *C. acutatum* isolate CECT 20240 conidial germination. **(B–F)** Photomicrographs of *C. acutatum* structures formed during infection of strawberry leaves over a 9-days period. **(B)** Non germinated conidia at 1 dpi; **(C)** Germinated conidia after 3 dpi; **(D,E)** Appressorium and mycelium development at 5 and 7 dpi, respectively; **(F)** Abundant mycelium at 9 dpi. All bars 25 μm.

To identify the initial infection stages, conidial development was monitored. The number of ungerminated conidia, germinated conidia without forming appresoria, and germinated conidia with fully developed appressoria, was monitored 1, 3, 5, 7 and 9 dpi (days post-inoculation; Figure 1). No visual disease symptoms were detected in the plant during this period of time. At 5 dpi 44.76% of conidia registered fully developed appresoria (Figure 1A). Other conidia did not germinate (34.98%); others, while they did, they did not produce appresoria (20.24%). Early mycelium formation was observed microscopically at 5 dpi (Figure 1D), whereas more extended and abundant mycelium was detected at 7 and 9 dpi, respectively (Figures 1E,F).

**Figure 2:**
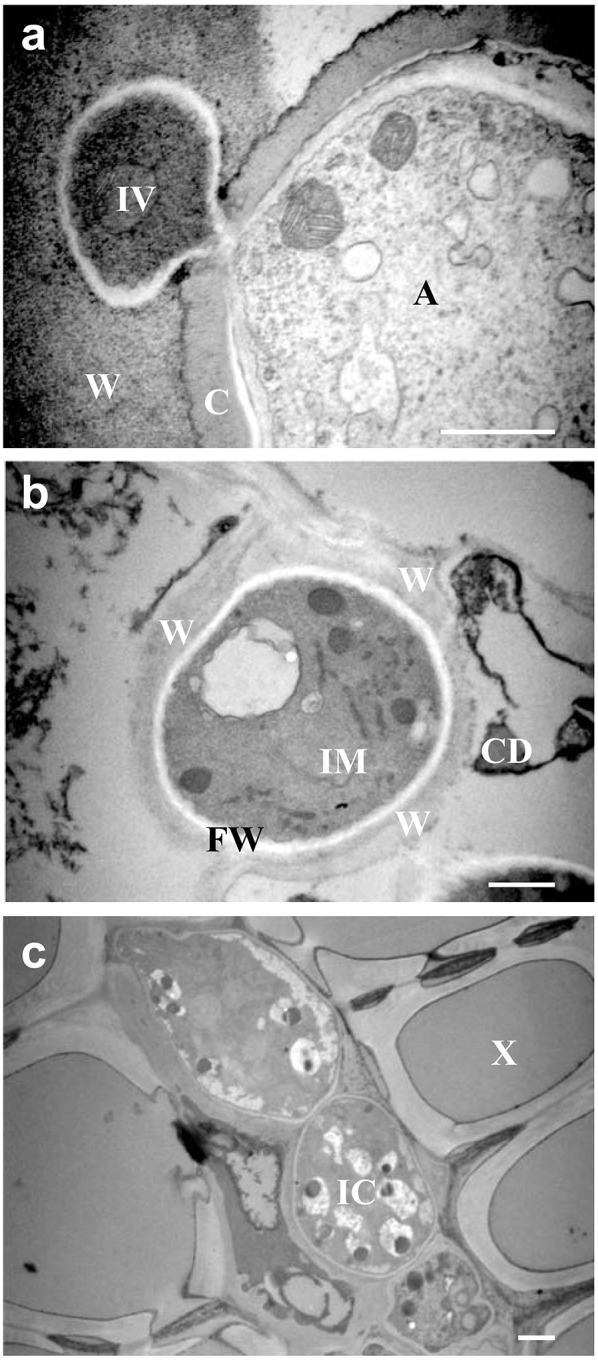
**Transmission Electronic Microscopy (TEM) in strawberry crown. (A)** Penetration of cuticle and biotrophic stage of *C. acutatum* by subcuticular hyphae on wall epidermal cell 36 h post-inoculation. **(B)** Necrotrophic signals of development of *C. acutatum* by intercellular hypha 4 days post-inoculation. **(C)** Necrotrophic development of *C. acutatum* by intracellular hyphae in xylem tissues at 7 dpi. A, appresorium; C, plant cuticle; IV, infection vesicle; W, wall plant cell; IM, intramural o intercellular hypha; FW, fungal cell wall; CD, cell debris; IC, intracellular hyphae; X, xylem. Bar = 1 μm.

As pathogen development in crown it is difficult to monitor by optical microscopy in the same way as aerial tissues, the progress of pathogen development in crown was monitored using TEM with the inoculation methods presented in Arroyo et al. (2005) (Figure 2). Such higher concentration of inoculum expedited infection development. Figure 2A shows penetration peg formation through the host cuticle and a small infection vesicle, 36 h post-inoculation (hpi), which reflects the establishment of the biotrophic stage of *C. acutatum*. No morphological signs of cuticular component degradation were observed during this penetration phase. Necrotic signals appeared as early as 4 dpi (Figure 2B), and pathogen development clearly expanded throughout vascular tissue at 7 dpi (Figure 2C).

### Oxidative stress analyses

**Figure 3:**
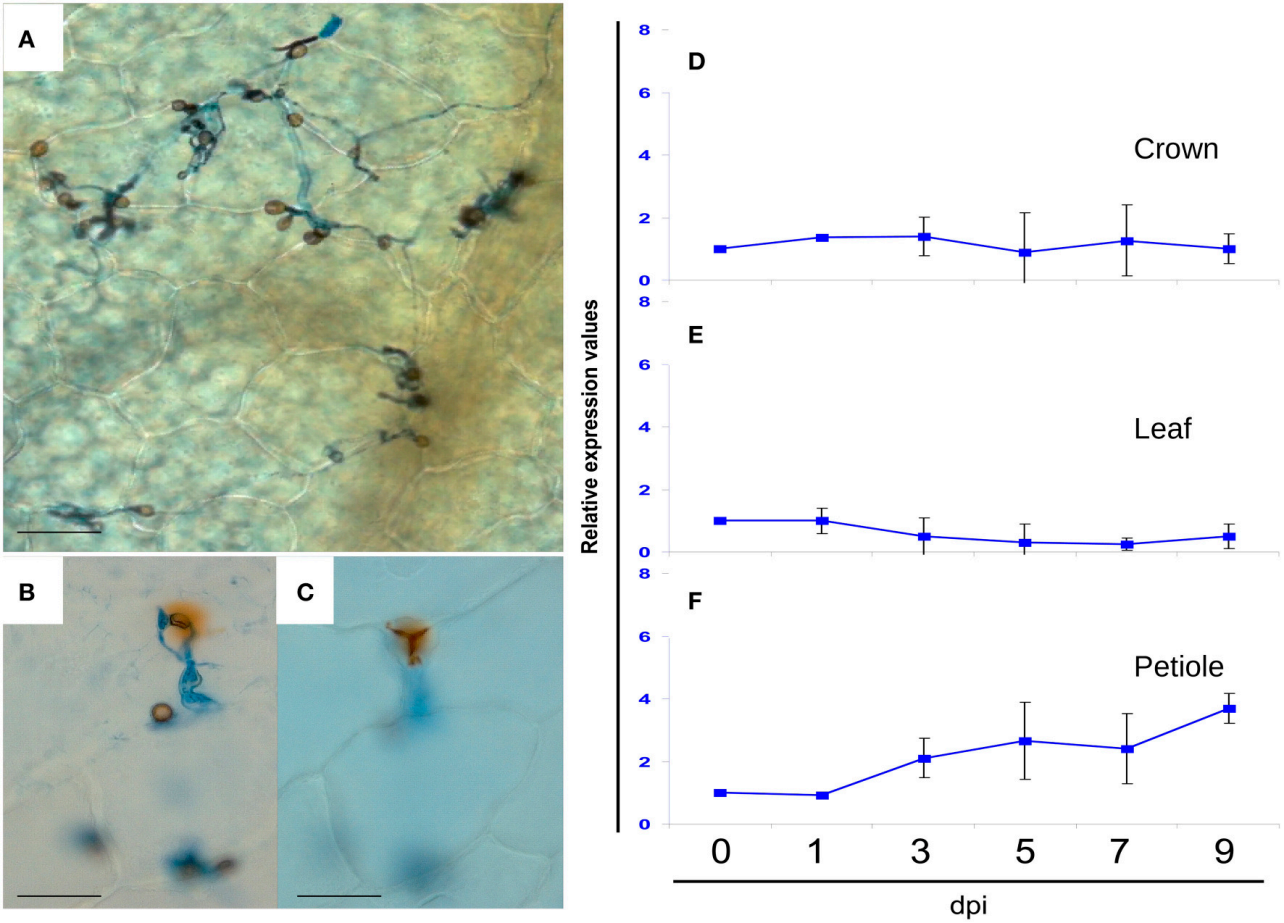
**Light microscopic analysis of DAB stained preparations of *C. acutatum* infected strawberry tissue at 7 dpi, and relative expression values by RTqPCR analysis of FaGST1 (At1g02930 ortholog) gene at different time points of infection. (A)** Tissue sample showing no DAB reaction, **(B,C)** very rarely detected reduction of DAB beneath fungal appressoria, and surrounding the penetration pegs, at upper and lower focal planes, respectively. All bars 25 μm. **(D–F)** Relative FaGST1 expression values. At each time point, every inoculated sample was compared with its corresponding mock treated sample. In the graphics, standard value 1 at T0 was added to better illustrate changes.

ROS production was monitored by diaminobenzidine (DAB) staining throughout the plant infection time-course. No clear accumulation of DAB (reddish-brown color visible to the naked-eye) was evident at any stage of the infection analyzed here (data not shown). Furthermore, under light microscopy, DAB staining beneath fungal appressoria and surrounding the penetration pegs was very rarely observed and only after 7 dpi (Figures 3A–C). FaGST1 transcripts (the strawberry AtGST1 orthologous gene) did not increase across the time points examined, neither in crown (Figure 3D) nor in leaves (Figure 3E). A slight increase in the expression of this gene was detected in petiole tissue 3–5 dpi (Figure 3F).

Based on these results, and the observation that spray inoculation with 10^4^ conidia·ml^−1^ more closely mimics natural infection conditions, the 5 dpi time point was chosen for transcriptome analysis. The assumption was that most of the changes in transcript accumulation in response to early *C. acutatum* infection would be detected around this time point, including those in response to early stages of switching to necrotrophic growth. In addition, analysis of known defense-related transcripts was performed at 1, 3, 5, 7, and 9 dpi to assess plant response to a wider range of fungal developmental stages and disease progression.

### Expression profiling of *C. acutatum*-infected strawberry

Transcript abundance was measured in crown tissue 5 days after *C. acutatum* infection and in mock-treated plants. Tables 1A,B and Tables S1, S2 show a summary of identified transcripts with the highest induction or repression levels after crown infection. A total of 147 transcripts varied in accumulation more than 1.75-fold following criteria described under Material and Methods. Of these, 118 genes were induced, and 29 genes were repressed. Verification of gene expression changes by real-time RTqPCR was performed on eleven up-regulated genes and two down-regulated genes, representing different categories shown in Tables 1A,B (marked with #). The expression pattern of analyzed genes after *C. acutatum* inoculation was consistent with that obtained by microarray analysis.

**Table 1A:** **Up-regulated genes by *Colletotrichum acutatum* in crown tissue of *Fragaria x ananassa*, cultivar Camarosa**.

***Fxananassa* gene ID**	***F. vesca* ortholog**	***A. thaliana* ortholog**	**Gene description**	**Relation with defense/biological function**	**CC vs. CI**	
					**Fold change (*RTqPCR*)**	**FDR *q*-value**
**INVASION SENSING**						
M13C5^*^	gene07245	AT5G13160	Serine/threonine-protein kinase PBS1	Receptor kinase, R protein-guard model	4.17	0
M19F7^*^	gene15497	AT4G33210	SLOMO (SLOw MOtion) F-box/LRR-repeat protein	Fbox/LRR protein, plant receptor, Proteasome complex	2.65	7.93E-03
M2F10^*^	gene19270^†^	AT4G00340	Receptor-like protein kinase 4	Receptor kinase, Signal transduction regulation	2.43	7.93E-03
M14D5	gene13911	AT1G30240	Proline-, glutamic acid- and leucine-rich protein 1	LRR protein, plant receptor	2.35	7.93E-03
M6C2	gene01890	AT5G42090	Lung seven transmembrane receptor family protein	Plant receptor	2.35	7.93E-03
ELRR-39#	gene25524	AT5G21090	CPR30 Leucine-rich repeat (LRR) family protein	LRR protein, plant receptor	2.14 (2.82 ± 0.54)	7.93E-03
M29F3	gene16731	AT3G14460	LRR and NB-ARC domains-containing disease resistance protein	CC-NBS-LRR class of R proteins, plant receptor	2.00	7.93E-03
M18E3	gene20858	AT3G14460	Proline-, glutamic acid- and leucine-rich protein 1	CC-NBS-LRR class of R proteins, plant receptor	1.84	7.93E-03
**SIGNAL TRANSDUCTION**						
M23A9	gene14522	AT4G35790	Phospholipase D delta	Phospholipase D, Transduction of stress responses	8.26	0
M27D3	gene18784	AT5G01160	RING/U-box E3 ubiquitin-protein ligase	E3 ligase, Proteasome complex	7.44	0
M16B7	gene00744	AT1G69960	Serine/threonine-protein phosphatase PP2A catalytic subunit	Ser/Thr protein phosphatase 2A catalytic subunit	5.20	0
M19D11	gene10418^†^	AT3G03940	Casein Serine/threonine-protein kinase	Calcium binding kinase	4.59	0
M13C5^*^	gene07245	AT5G13160	Serine/threonine-protein kinase PBS1	Receptor kinase, R protein-guard model	4.17	0
EDS1-936^*^#	gene09503	AT3G48090	EDS1-specific diacylglycerol lipase alpha	Lipase, SA pathway regulator	3.82 (4.4 ± 1.13)	0
M23A6	gene32391	AT4G11740	Ubiquitin-like superfamily protein	Ubiquitin, Proteasome complex	3.49	7.60E-03
M27C10	gene30942_.3utr_	AT5G25510	Serine/threonine protein phosphatase 2A regulatory B subunit	Ser/Thr protein phosphatase 2A catalytic subunit	3.44	0
M8G2	gene10067	AT4G30960	CIPK-Serine/threonine-protein kinase 6	Calcium binding kinase SOS2	3.18	0
M4F10	gene21532	AT1G65430	E3 ubiquitin-protein ligase ARI8	E3 ligase, Proteasome complex	3.15	0
M8G7	gene24036	AT4G36990	TBF1 Heat shock factor protein	Major molecular switch for plant growth-to-defense transition	3.06	7.93E-03
M24D7^*^	gene28350	AT5G40150	Peroxidase superfamily protein	Class III peroxidase	2.76	7.93E-03
M10E2	gene02575	AT1G27460	NPGR1-No pollen germination related 1	Calmoduling binding protein	2.73	7.93E-03
M3D5	gene23778	AT1G05180	NEDD8-activating enzyme E1 regulatory subunit / AXR1 (Auxin resistant 1) NAD(P)-binding Rossmann-fold superfamily protein	Fbox, JA signaling, Proteasome complex	2.73	7.93E-03
M19F7^*^	gene15497	AT4G33210	SLOMO (SLOw MOtion) F-box/LRR-repeat protein	Fbox/LRR protein, plant receptor, Proteasome complex	2.65	7.93E-03
M25E7	gene01516	AT1G15780	Bromodomain-containing protein	Interact with calcium binding protein kinase	2.47	7.93E-03
M8D11^*^	gene06214	AT1G60490	Phosphatidylinositol 3-kinase	Protein kinase, Protein trafficking, Secretory Pathway	2.46	7.93E-03
M13H9	gene12681	AT5G57020	Myristoyl-CoA:protein N-myristoyltransferase	Co-traslational addition of myristic acid	2.45	7.93E-03
M2F10^*^	gene19270^†^	AT4G00340	Receptor-like protein kinase 4	Receptor kinase, Signal transduction regulation	2.43	7.93E-03
M7G11	gene04753	AT1G69640	Sphingoid base hydroxylase 1 (SBH1)	Synthesis of membrane components	2.42	7.93E-03
M4E10^*^#	gene16110	AT3G52430	Phytoalexin deficient 4, Lipase	Lipase, Chemical defenses, SA pathway regulator	2.33 (2.67 ± 0.64)	7.93E-03
M4C3	gene15015	AT5G10930	CIPK-Serine/threonine-protein kinase 5	Calcium binding kinase	2.25	7.93E-03
M14H1	gene07894	AT3G51860	Vacuolar cation/proton exchanger 3	Proton/Calcium antiporter	2.20	7.93E-03
M7B6	gene05859	AT1G80210	BRCC36A - homologous recombination	Homologous recombination, deubiquitinating activity, Proteasome complex	2.20	7.93E-03
M21H5	gene01441	AT5G56180	Actin-related protein 8	Fbox/Actin/helicase domain, proteasome complex, XXXRNAmetabolism	2.05	7.93E-03
M4E6	gene12959	AT4G33240	1-phosphatidylinositol-4-phosphate 5-kinase	Protein kinase, protein trafficking, endomembrane homeostasis	2.04	7.93E-03
M28C8^*^	gene12445	AT1G05260	Peroxidase superfamily protein	Class III peroxidase	1.98	7.93E-03
M17E3	gene06367^†^	AT4G24830	Argininosuccinate synthase	NO synthesis, signal transduction	1.98	7.93E-03
M3E6^*^#	gene27591	AT1G71695	Peroxidase superfamily protein (Prx12)	Class III peroxidase	1.92 (5.5 ± 0.7)	7.93E-03
M10B6	gene01594	AT3G13460	YTH domain family protein 2	Calcium transport to nucleus, regulate gene expression	1.86	7.93E-03
M13F3	gene28416	AT3G27925	Protease DegP1	Protease	1.79	7.93E-03
M1H8	gene12874^†^	AT5G53360	E3 Ubiquitin protein ligase SINAT3	E3 ligase, proteasome complex	1.75	9.42E-03
**NEW PROTEIN SYNTHESIS AND SECRETION**						
M21B3#	gene01340	AT5G13080	WRKY DNA-binding protein 75	Transcription factor	5.79 (19.47 ± 5.2)	0
M8H8	gene10702	AT4G17960	ATP-dependent RNA helicase DBP10	RNA metabolism	5.61	0
M26G7	gene31909	AT2G25970	RNA binding KH domain-containing protein	RNA metabolism	5.35	0
M22D9	gene22758	AT3G51980	Armadillo repeat superfamily protein-Hsp70 nucleotide exchange factor fes1	Protein folding	4.80	0
J_4-9#	gene07210	AT5G13080	WRKY DNA-binding protein 75	Transcription factor	3.89 (56.68 ± 7.93)	0
M11C6	gene03828	AT1G69620	60S Ribosomal protein L34	Protein synthesis	3.79	0
M6G7	gene32154	AT3G48030	Hypoxia-responsive Zinc finger (C3HC4-type RING finger) family protein	Transcription factor	3.75	0
M10C12	gene08531	AT1G75780	Tubulin beta-1 chain	Citosqueleton	3.62	0
M9F6	gene29752	AT1G28420	Homeobox protein orthopedia	Transcription factor	3.44	0
M1A2	gene24354	AT1G62020	Coatomer subunit alpha	Protein transport	3.31	0
M23C4	gene02623	AT4G37750	AINTEGUMENTA gene - AP2 like transcription factor	Transcription factor	3.20	7.60E-03
M18A9	gene30367	AT5G46190	RNA-binding KH domain-containing protein	RNA metabolism	2.85	7.93E-03
M7G4	gene23202	AT3G52250	Duplicated homeodomain-like superfamily protein	RNA metabolism	2.75	7.93E-03
M23C7	gene25539_.3utr_	AT4G33865	40S ribosomal protein S29	Protein synthesis	2.57	7.93E-03
M17H1^*^#	gene13547	AT3G56400	WRKY DNA-binding protein 70	Transcription factor, SA-JA crosstalk	2.53 (4.99 ± 0.54)	7.93E-03
M18F1	gene09051	AT1G47490	RNA-binding protein 47C	RNA metabolism	2.49	7.93E-03
M8D11^*^	gene06214	AT1G60490	Phosphatidylinositol 3-kinase	Protein kinase, Protein trafficking, Secretory Pathway	2.46	7.93E-03
M11H4	gene22626	AT3G12110	Actin 11	Citosqueleton	2.42	7.93E-03
M8H3^*^#	gene13803	AT2G38470	WRKY DNA-binding protein 33	Transcription factor, JA pathway	2.41 (3.58 ± 1.52)	7.93E-03
M14B5	gene29081	AT1G59740	Peptide transporter PTR	Protein secretion	2.39	7.93E-03
M5B8	gene24582	AT5G22950	Vacuolar protein sorting-associated protein 24	Protein secretion	2.22	7.93E-03
M12E12^*^#	gene21365	AT3G56400	WRKY DNA-binding protein 70	Transcription factor, SA-JA crosstalk	2.19 (2.96 ± 0.54)	7.93E-03
M3A1	gene30880	AT3G16060	Kinesin-related protein	Citosqueleton	2.12	7.93E-03
M19E4	gene05323	AT2G44710	RNA-binding (RRM/RBD/RNP motifs) family protein	RNA metabolism	2.12	7.93E-03
M18C5	gene04135	AT1G66140	Zinc finger protein 4	Transcription factor	2.08	7.93E-03
M3E11	gene25805	AT1G18650	Plasmodesmata callose-binding endo-1,3-beta-glucosidase protein 3 (PdCB3)	Cell-to-cell trafficking	2.02	7.93E-03
M12B6	no hit found^†^	AT3G25940	DNA-directed RNA polymerase TFIIB zinc-binding protein	RNA metabolism	2.01	7.93E-03
M7D1	gene10625	AT3G05590	60S ribosomal protein L18-2	Protein synthesis	2.00	7.93E-03
M20A3	gene21473	AT5G16715	Valyl-tRNA synthetase	Protein synthesis	1.98	7.93E-03
M8A6	gene00998	AT1G77030	DEAD-box ATP-dependent RNA helicase 29	RNA metabolism	1.93	7.93E-03
M9E2	gene15731	AT1G80070	Pre-mRNA-processing-splicing factor SUS2	RNA metabolism	1.92	7.93E-03
M28B7	gene16235_.5utr_	AT2G22430	Homeobox-leucine zipper protein ATHB-6	Transcription factor	1.89	7.93E-03
M1C12^*^#	gene28174	AT2G38470	WRKY DNA-binding protein 33	Transcription factor, JA pathway	1.86 (9.10 ± 1)	7.93E-03
M6A9	gene00185	AT5G67300	Transcription factor MYB44	Transcription factor	1.83	9.42E-03
M4C6	gene20572	AT3G62310	RNA helicase family protein	RNA metabolism	1.79	9.42E-03
**DIRECT DEFENSES**						
M24B7#	gene14817	AT4G16260	Glycosyl hydrolase superfamily protein	Cell wall degradation, PR protein family	47.54 (30.54 ± 16.25)	0
M16D12#	gene02717	AT3G54420	Chitinase class IV	PR protein family	7.93 (116.84 ± 22.54)	0
EPR5-77#	gene32423	AT4G11650	Pathogenesis-related 5 family protein	PR protein family	7.52 (59.11 ± 10.05)	0
M5B6	gene24296_.3utr_	AT5G09360	Laccase	Lignin biosynthesis	7.48	0
M23A10	gene07086	AT1G24020	Pathogenesis-related 10 family protein	PR protein family	7.08	0
M12C12#	gene31975	AT5G14180	Triacylglycerol lipase 2	Lipase, Chemical defenses	6.60 (19.59 ± 2.7)	0
M6G11	gene26351	AT4G34135	Flavonol 7-O-glucosyltransferase	Secondary metabolism	4.34	0
M6B9	gene05185	AT1G24020	Pathogenesis-related 10 family protein	PR protein family	3.89	0
EPR5-284#	gene32422	AT4G11650	Pathogenesis-related 5 family protein	PR protein family	3.88 (5.63 ± 1.93)	0
M1F10#	gene09812	AT1G20030	Pathogenesis-related 5 family protein	PR protein family	3.69 (17.58 ± 5.7)	0
M22A10#	gene07085	AT1G24020	Pathogenesis-related 10 family protein	PR protein family	3.20 (8.87 ± 2.38)	0
M24D7^*^	gene28350	AT5G40150	Peroxidase superfamily protein	Class III peroxidase	2.76	7.93E-03
M5G8	gene07082	AT1G24020	Pathogenesis-related 10 family protein	PR protein family	2.67	7.93E-03
M10C5	gene00687	AT1G24020	Pathogenesis-related 10 family protein	PR protein family	2.66	7.93E-03
M26E5	gene32023	AT5G17000	Zinc-binding dehydrogenase family protein / oxidoreductase	Redox protection	2.65	7.93E-03
M4F3	gene27555	AT1G22750	D-serine/D-alanine/glycine transporter	Secondary metabolism	2.65	7.93E-03
M25D10	gene07087	AT1G24020	Pathogenesis-related 10 family protein	PR protein family	2.44	7.93E-03
M5C8	gene11632	AT4G32320	L-ascorbate peroxidase 6	Antioxidant defenses	2.36	7.93E-03
M4E10^*^	gene16110	AT3G52430	Phytoalexin deficient 4, Lipase	Lipase, Chemical defenses, SA pathway regulator	2.33 (2.67 ± 0.64)	7.93E-03
M23D11	gene20700	AT4G37990	Cinnamyl alcohol dehydrogenase	Lignin biosynthesis	2.14	7.93E-03
M29A9	gene21697	AT3G54420	Endochitinase PR4	PR protein family	2.02	7.93E-03
M25D11	gene17437	AT3G07320	O-Glycosyl hydrolases family 17, (1->3)-beta-glucanase	Cell wall degradation, PR protein family	1.98	7.93E-03
M28C8^*^	gene12445	AT1G05260	Peroxidase superfamily protein	Class III peroxidase	1.98	7.93E-03
M3E6^*^#	gene27591	AT1G71695	Peroxidase superfamily protein (Prx12)	Class III peroxidase	1.92 (5.5 ± 0.7)	7.93E-03
M10D7	gene07065	AT1G24020	Fra a 2 allergen	PR protein family	1.86	7.93E-03
M26G2	gene31048	AT2G30370	CHAL secreted protein	Inhibite stomatal production	1.79	7.93E-03
M21G5	gene04724	AT1G69530	Expansin-A1	Stomatal movement	1.76	9.42E-03
**HORMONE-DEPENDENT PATHWAYS**						
EDS1-936^*^#	gene09503	AT3G48090	EDS1-specific diacylglycerol lipase alpha	Lipase, SA pathway regulator	3.82 (4.4 ± 1.13)	0
M12E4	gene32179	AT1G27500	Tetratricopeptide repeat (TPR)-like superfamily protein	Tetratricopeptide repeat	3.32	0
M22A6	gene05545	AT1G80360	Pyridoxal phosphate (PLP)-dependent transferases superfamily protein	Pyridoxal-phosphate, oxidative stress response	2.84	7.93E-03
M14G2	gene31738	AT4G39820	Tetratricopeptide repeat (TPR)-like superfamily protein	Tetratricopeptide repeat	2.69	7.93E-03
M8H2	gene09899	AT5G64250	2-nitropropane dioxygenase	JA pathway	2.67	7.93E-03
M26D3	gene18908	AT4G01100	Adenine nucleotide transporter 1 (ADNT1)	Purine transporter, Signaling	2.56	7.93E-03
M17H1^*^#	gene13547	AT3G56400	WRKY DNA-binding protein 70	Transcription factor, SA-JA crosstalk	2.53 (4.99 ± 0.54)	7.93E-03
M25B1	gene23034	AT3G13790	Cell wall Invertase 1 (AtcwINV1): Glycosyl hydrolases family 32 protein	Cell wall invertase, signaling	2.48	7.93E-03
M9E10	gene03078	AT1G44750	Purine permease 11	Purine transporter, Signaling	2.44	7.93E-03
M8H3^*^#	gene13803	AT2G38470	WRKY DNA-binding protein 33	Transcription factor, JA pathway	2.41 (3.58 ± 1.52)	7.93E-03
M4E10^*^#	gene16110	AT3G52430	Phytoalexin deficient 4, Lipase	Lipase, Chemical defenses, SA pathway regulator	2.33 (2.67 ± 0.64)	7.93E-03
M12E12^*^#	gene21365	AT3G56400	WRKY DNA-binding protein 70	Transcription factor, SA-JA crosstalk	2.19 (2.96 ± 0.54)	7.93E-03
M23C11	gene08617	AT1G76180	Dehydrin cold-regulated 47	ABA responsive	2.15	7.93E-03
M16H1	gene14094_.3utr_	no hit found	Auxin response factor	Auxin responsive	2.14	7.93E-03
M9D5	gene29393	AT4G37150	Methyl salicylate (MeSA) esterase 9	SA release from MeSA	2.03	7.93E-03
M30F8#	gene29769_.3utr_	AT1G28480	Glutaredoxin GRX480	SA pathway, REDOX signaling	1.92 (4.52 ± 0.82)	7.93E-03
M1C12^*^#	gene28174	AT2G38470	WRKY DNA-binding protein 33	Transcription factor, JA pathway	1.86 (9.10 ± 1)	7.93E-03
M28A2#	gene15063	AT5G42650	Allene oxide synthase	JA synthesis	1.75 (1.5 ± 0.64)	9.42E-03
**NO OBVIOUSLY RELATED TO DEFENSE RESPONSE**						
M22B1	gene01044	AT2G25660	Embryo defective 2410		8.25	0
M18E11	gene27435	AT1G34550	Embryo defective 2756		6.55	0
M21E9	gene24023	AT2G24960	MRG family protein, chromatin binding		3.31	0
M7B12	gene07388	AT2G21170	Triosephosphate isomerase		2.96	7.60E-03
M24C11	gene32086	AT1G64385	Unknown protein, endomembrane system		2.88	7.60E-03
M13A4	gene23331	AT5G13520	Aminopeptidase M1 family protein / Leukotriene A-4 hydrolase		2.39	7.93E-03
M27A2	gene13677^†^	AT1G32060	Phosphoribulokinase		2.08	7.93E-03
M4E4	gene05017^†^	AT5G49930	Embryo defective 1441		2.07	7.93E-03
M25G5	gene06563_.3utr_	AT4G13930	Serine hydroxymethyltransferase 4		2.04	7.93E-03
M3F5	gene13777	AT3G08890	Protein of unknown function		1.97	7.93E-03
M22G7	gene09933_.3utr_	AT5G41835	non-LTR retrotransposon family		1.93	3.64E-02
M4F8	gene15022_.3utr_	AT2G25140	Casein lytic proteinase B4/heat shock protein		1.91	7.93E-03

Genes were considered as differentially expressed if they fulfilled a FDR < 0.05 after a SAM test analysis and the fold-change was higher that 1.75-fold between the compared conditions. Fold change values represent the ratio of cv. Camarosa mock (CC) vs. infected (CI). (#) indicates genes further analyzed by real time RTqPCR to validate microarray result. Their relative expression value at 5dpi is shown as (media ± SD) in the “Fold Change” column. (

† *) marks no obvious detection of F. vesca ortolog gene due to putative fails by automated gene prediction (see also Table S3). 3utr, and 5utr, indicate F x ananassa sequences matching untranslated regions of the corresponding F. vesca gene (see also Table S3). Regulated genes are grouped in sections accordingly to their role in different steps of the defense response against C. acutatum (see Table S4 for associated references). Asterisk marks genes which take part in more than one unique functional group*.

**Table 1B:** **Down-regulated genes by *Colletotrichum acutatum* in crown tissue of *Fragaria x ananassa*, cultivar Camarosa**.

***Fxananassa* gene ID**	***F. vesca* ortholog**	***A. thaliana* ortholog**	**Gene Description**	**Relation with defense/biological function**	**CC vs. CI**	
					**Fold Change**	**FDR *q*-value**
**INVASION SENSING**						
M6F8	gene29223	AT1G57680	G-Protein coupled receptor 1	G-protein coupled receptor	−1.99	3.95E-02
M20C3	gene24345	AT2G32240	Leucine-rich repeat-containing protein	LRR protein, plant receptor	−1.93	3.95E-02
**SIGNAL TRANSDUCTION**						
M18F3	gene21849	AT5G43010	Regulatory particle AAA-ATPase 4A/Proteasome complex	Regulatory ATPase, Proteasome complex	−2.02	3.95E-02
M29G3#	gene25430	AT2G22990	Serine carboxypeptidase	Peptidase, Glucosinolate and phenylpropanoid pathway	−1.88 (−5.1 ± 2.2)	3.95E-02
M5E3	gene12921	AT1G74960	Beta-ketoacyl-ACP synthase	Fatty acid biosynthesis	−1.80	3.95E-02
M26F4	gene09121	AT5G67090	Subtilisin-like serine endopeptidase	Peptidase	−1.78	3.95E-02
M22F5	gene18417	AT5G02310	Protein ubiquitination component of the N-end rule	Ubiquitin ligase, Proteasome complex	−1.76	3.95E-02
**NEW PROTEIN SYNTHESIS AND SECRETION**						
M10H10	gene17514	AT2G32700	LEUNIG_homolog transcriptional correpresor	Transcription represor	−2.39	3.95E-02
M28F7	gene25662^†^	AT5G02960	40S Ribosomal protein S12/S23	Protein synthesis	−2.15	3.95E-02
M22E3	gene12861	AT5G53430	Histone methyltransferase	Indirect transcription regulation	−1.86	3.95E-02
M22E11	gene15974_.3utr_	AT1G15750	TOPLESS transcriptional correpresor	Transcription represor	−1.85	3.95E-02
M22D5	gene31183_.3utr_	AT1G22910	RNA-binding (RRM/RBD/RNP motifs) family protein	RNA metabolism	−1.78	3.95E-02
M21G2	gene29663	AT1G29170	SCAR family member	Citoesqueleton	−1.75	3.95E-02
**DIRECT DEFENSES**						
M29H6#	gene32347	AT4G22880	Leucoanthocyanidin dioxygenase (LDOX)	Secondary metabolism	−1.91 (−4.3 ± 2.11)	3.95E-02
M21F3	gene11045	AT1G36370	Serine hydroxymetyltransferase	REDOX production	−1.90	3.95E-02
M29C12	gene21346	AT5G05270	Chalcone-flavanone isomerase	Secondary metabolism	−1.89	3.95E-02
M19C6	gene26641	AT5G15870	Glycosyl hydrolase family 81 protein	Cell wall degradation, PR protein family	−1.76	3.95E-02
**HORMONE-DEPENDENT PATHWAYS**						
M18H1	gene14092	AT1G07590	Tetratricopeptide repeat (TPR)-like superfamily protein	Tetratricopeptide repeat	−1.82	3.95E-02
M15G5	gene02397	AT4G03550	Glucan / Callose synthase	Negative regulator SA dependent defenses	−1.80	3.95E-02
**NO OBVIOUSLY RELATED TO DEFENSE RESPONSE**						
M8D2	gene14995	AT5G17920	Methionine synthase		−2.20	3.95E-02
M9F8	gene16275	AT4G39970	Haloacid dehalogenase-like hydrolase		−2.02	3.95E-02
M7B2	gene10408	AT3G03890	Flavin mononucleotide binding		−1.94	3.95E-02
M14A10	gene29476	AT5G52820	WD-40 repeat CUL4 RING ubiquitin ligase complex		−1.94	3.95E-02
M5B7	gene09169	AT1G48380	DNA binding protein ROOT HAIRLESS 1, component of the topoisomerase VI complex		−1.92	3.95E-02
M18D12	gene20804	AT2G22530	Alkaline-phosphatase-like family protein		−1.83	3.95E-02
M18A11	gene08921	AT5G47470	Nodulin transporter family protein		−1.83	3.95E-02
M28A7	gene15006	AT5G10840	Endomembrane protein 70 protein family		−1.81	3.95E-02
M26H5	gene18624	AT1G01090	Pyruvate dehydrogenase alpha		−1.78	3.95E-02
M11B2	gene07537	AT3G13990	Kinase-related protein		−1.76	3.95E-02

Genes were considered as differentially expressed if they fulfilled a FDR < 0.05 after a SAM test analysis and the fold-change was higher that 1.75-fold between the compared conditions. Fold change values represent the ratio of cv. Camarosa mock (CC) vs. infected (CI), transformed by: -1/fold-change for better understanding of values. (#) indicates genes further analyzed by real time RTqPCR to validate microarray result. Their relative expression value at 5dpi is shown as (media ± SD) in the “Fold Change” column. (

† *) marks no obvious detection of F. vesca ortolog gene due to putative fails by automated gene prediction (see also Table S3). 3utr, and 5utr, indicate F × ananassa sequences matching untranslated regions of the corresponding F. vesca gene (see also Table S3). Regulated genes are grouped in sections accordingly to their role in different steps of the defense response against C. acutatum (see Table S4 for associated references)*.

Automated functional analysis by FunCat and GO terms assignments showed that many of these up- and down-regulated genes described in Tables 1A,B belong to plant defense and stress response-related categories (Figure S1 and Tables S3–S5). However, when no obvious functional role was annotated within the corresponding *F. vesca* ortholog genes, a thorough search through the references available in the database from many plant species was performed (see Table S3). A wider range of the strawberry transcripts matched defense and biotic stress annotations with the number of up- and down-regulated genes changing 89.93 and 65.51%, respectively.

Transcripts representing five subsets of putative molecular function were identified. These include plant receptors, signal transduction mechanisms under hormonal control (protein modification and degradation), transcriptional changes (transcription factors), new protein synthesis, and secretion of active defense components (PR proteins, degradative enzymes or chemical defenses).

#### Singular enrichment analysis (SEA) shows key components of SA-mediated signaling pathway are up-regulated

**Figure 4:**
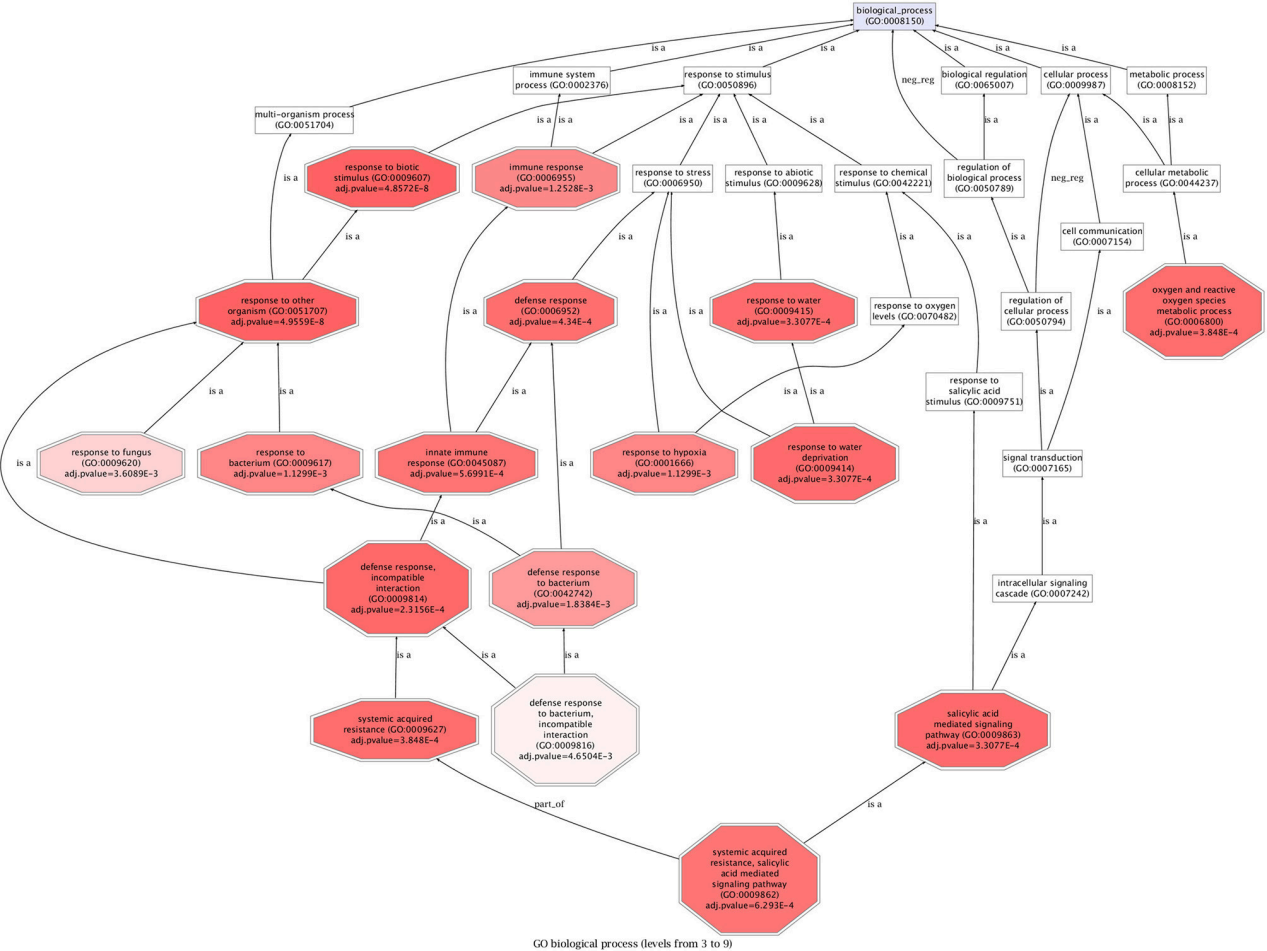
**Singular Enrichment Analysis (SEA) on strawberry up-regulated genes**. FatiGO was used to extract relevant GO terms for biological processes (BP) at *p* < 0.005. The terms are considered to be relevant by the application of statistical tests as described in Al-Shahrour et al. (2004). Data are presented as a heat map, as prompts color intensity correlates with adj. *p*-value, the highest intensity, the lowest adj. *p*-value. See Table S1 for a detailed list of further genes belonging to overrepresented functions.

A comprehensive Singular Enrichment Analysis (SEA) using FatiGO (Al-Shahrour et al., 2004) identified key processes altered in strawberry after *C. acutatum* infection (Figure 4, and Table S6). Transcripts increasing in abundance include (*p* < 0.005) those associated with Systemic Acquired Resistance and SA-mediated signaling pathways, responding to bacterium and fungus, and activating the immune response. Strawberry genes within these enriched categories are genes FaEDS1 (EDS1-936^EST^, AtEDS1-like) and FaPAD4 (M4E10^EST^, AtPAD4-like), which are known to be involved in PRR- and R-mediated pathogen-induced SA accumulation in other plants; genes FaWRKY70-1 and FaWRKY70-2 (M17H1^EST^, and M12E12^EST^, respectively, two AtWRKY70-like genes); gene FaMeSA1 (M9D5^EST^, a methyl salicylate esterase); gene FaPBS1 (M13C5^EST^, a SA-dependent Ser/Thr kinase); and gene FaGRX1 (M30F8^EST^, similar to a member of the glutaredoxin family that regulates the protein redox state), which are major downstream components of the SA signal transduction pathway, and known to be activators of SA-dependent defense in many plants.

#### RTqPCR analysis indicates that components of jasmonic acid defense signaling pathway are also induced in strawberry after C. acutatum infection

**Figure 5:**
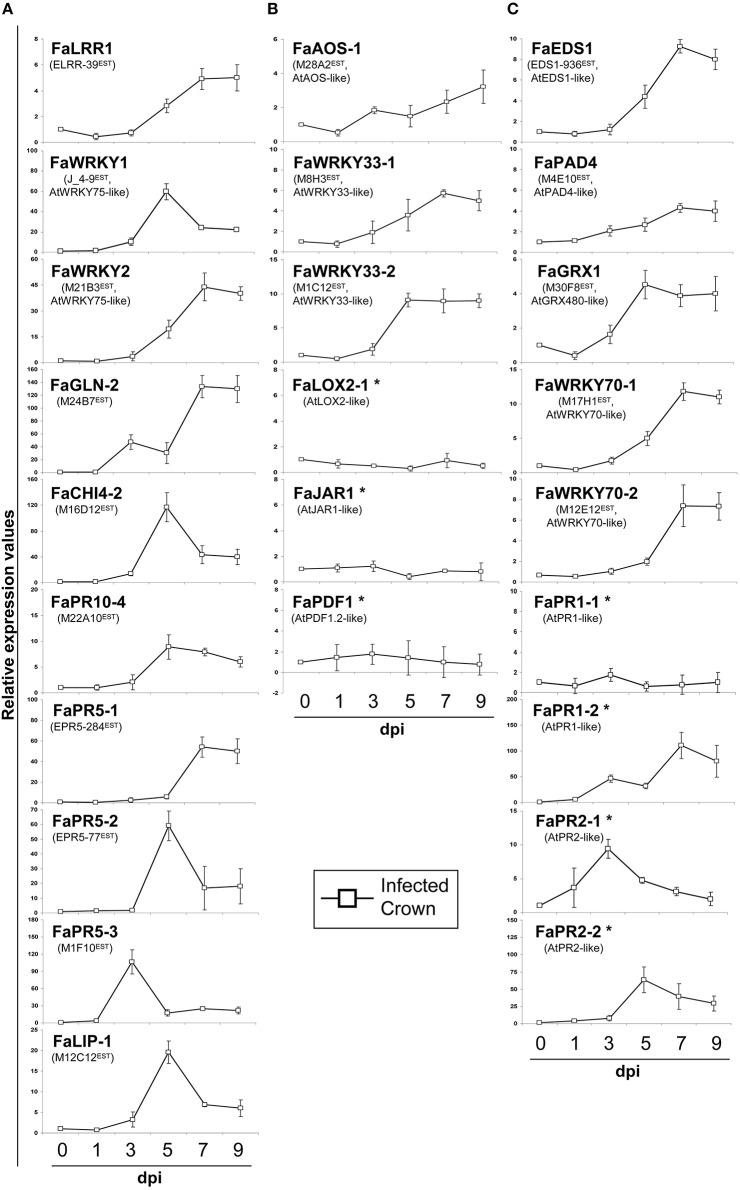
**Relative expression values by RTqPCR analysis of upregulated strawberry genes during *C. acutatum* infection. (A)** Relevant strawberry genes in this study, **(B)** JA-responsive marker genes, and **(C)** SA-responsive marker genes. Strawberry crowns were harvested at different days post-treatment (dpi) either with mock or *C. acutatum* spore suspension. At each time point, every inoculated sample was compared with its corresponding mock treated sample. In the graphics, standard value 1 at T0 was added to better illustrate changes. Asterisk marks genes not present in the Array dataset. Arabidopsis orthologous genes are *AT5G13080* (*AtWRKY75*), *AT5G42650* (*AtAOS*), *AT2G38470* (*AtWRKY33*), *AT3G45140* (*AtLOX2*), *AT2G46370* (*AtJAR1*), *AT5G44420* (*AtPDF1.2*), *AT3G48090* (*AtEDS1*), *AT3G52430* (*AtPAD4*), *AT1G28480* (*AtGRX480*), *AT3G56400* (*AtWRKY70*), *AT2G14610* (*AtPR1*), *AT3G57260* (*AtPR2*).

Analysis of a representative set of up-regulated genes, including those used for microarray validation (see Tables 1A,B), was carried out by RTqPCR after crown infection (Figure 5). All genes tested showed clear upregulation after infection (Figure 5A). Similar results were also found on petiole tissue analysis after infection (Figure S2A). In addition, two main expression patterns were detected. Transcripts corresponding to FaWRKY1 (J_4-9^EST^), FaLIP-1 (M12C12^EST^), FaCHI4-2 (M16D12^EST^), FaPR5-2 (EPR5-77^EST^), and FaPR10-4 (M22A10^EST^) reached a maximum level at 5 dpi, while transcripts corresponding to FaWRKY2 (M21B3^EST^), FaLRR1 (ELRR-39^EST^), FaGLN-2 (M24B7^EST^), and FaPR5-1 (EPR5-284^EST^) reached their maximum expression level at later times. Transcripts representing FaWRKY1 (J_4-9^EST^) and FaWRKY2 (M21B3^EST^), which belong to the same family of transcription factors (orthologs to Arabidopsis AtWRKY75), registered a different timing in their response. Other variations in timing were also detected for the three members of the PR5 family (FaPR5-1, FaPR5-2, FaPR5-3).

**Figure 6:**
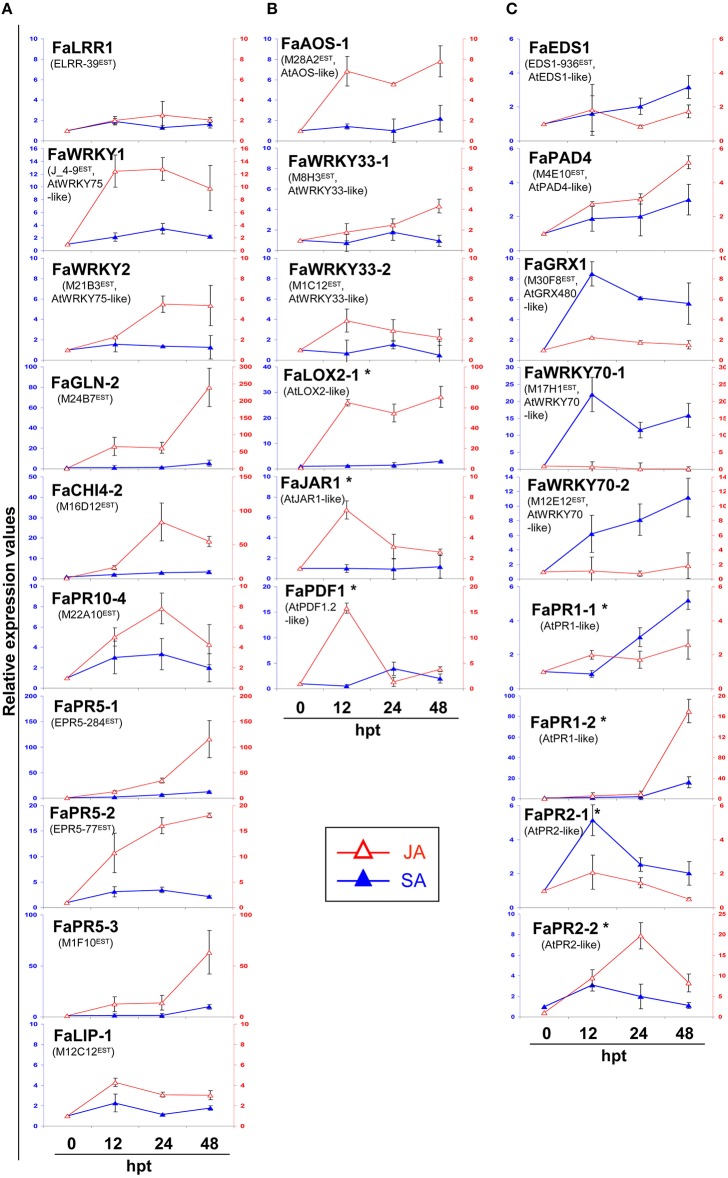
**Relative expression values by RTqPCR analysis of upregulated strawberry genes by hormone treatment. (A)** Relevant strawberry genes in this study, **(B)** JA-responsive marker genes, and **(C)** SA-responsive marker genes. Strawberry plantlets were treated with mock, SA (5 mM) and MeJA (2 mM) elicitors, and harvested at different hours post-treatment (htp). At each time point, every elicited sample was compared against its corresponding mock treated sample. Left and right legends represent relative expression values for SA and JA treatments, respectively. In the graphics, standard value 1 at T0 was added to better illustrate changes. Asterisk marks genes not present in the Array dataset. Arabidopsis orthologous are *AT5G13080* (*AtWRKY75*), *AT5G42650* (*AtAOS*), *AT2G38470* (*AtWRKY33*), *AT3G45140* (*AtLOX2*), *AT2G46370* (*AtJAR1*), *AT5G44420* (*AtPDF1.2*), *AT3G48090* (*AtEDS1*), *AT3G52430* (*AtPAD4*), *AT1G28480* (*AtGRX480*), *AT3G56400* (*AtWRKY70*), *AT2G14610* (*AtPR1*), *AT3G57260* (*AtPR2*).

An additional experiment was performed using *in-vitro* plantlets to describe networks associated with SA or JA treatment. Plantlets were used because they show enhanced sensitivity and a faster response to hormone treatment against mature plants (Figure 6). Almost all *C. acutatum* induced transcripts also showed significant induction after MeJA, but not so after SA treatment (Figure 6A). Transcripts corresponding to PR genes such as FaGLN-2 (M24B7^EST^), FaCHI4-2 (M16D12^EST^), FaPR5-2 (EPR5-77^EST^), FaPR5-1 (EPR5-284^EST^), FaPR5-3 (M1F10^EST^), and FaPR10-4 (M22A10^EST^), as well as the WRKY75-like transcription factors [genes FaWRKY1 (J_4-9^EST^) and FaWRKY2 (M21B3^EST^)] increased in abundance in response to JA.

### Incomplete activation of SA and JA pathways occurs during *C. acutatum* infection

To further investigate how SA- and JA- hormone-dependent pathways are responding to *C. acutatum* infection, transcript levels of their described signaling pathways were measured by RTqPCR. Transcripts corresponding to JA-associated genes FaWRKY33-1 (M8H3^EST^) and FaWRKY33-2 (M1C12^EST^) (two orthologs to AtWRKY33), FaAOS-1 (M28A2^EST^, AtAOS ortholog), FaLOX2-1 (AtLOX2 ortholog), FaJAR1 (AtJAR1 ortholog), and FaPDF1 (AtPDF1.2 ortholog), and SA-associated transcripts such as FaEDS1 (EDS1-936^EST^, AtEDS1 ortholog), FaPAD4 (M4E10^EST^, AtPAD4 ortholog), FaGRX1 (M30F8^EST^, AtGRX480 ortholog), FaWRKY70-1 and FaWRKY70-2 (M17H1^EST^, and M12E12^EST^, respectively), FaPR1-1 and FaPR1-2 (AtPR1 ortholog), FaPR2-1 and FaPR2-2 (AtPR2 ortholog), were analyzed in crown tissue in response to *C. acutatum* inoculation (Figures 5B,C), and after MeJA or SA exogenous applications (Figures 6B,C).

None of known JA inducible pathways, except for gene regulators FaWRKY33-1, FaWRKY33-2, and FaAOS-1 (whose induction was also detected by microarray, see Tables 1A,B) were infection induced (Figure 5B). Similar results were also obtained on petiole tissue analysis after infection (Figure S2B). On the contrary, all transcripts were induced (FaAOS-1, FaWRKY33-1, FaWRKY33-2, FaLOX2-1, FaJAR1, and FaPDF1) in response to MeJA treatment (Figure 6B).

On the other hand all SA-pathway associated orthologs were induced in crown after *C. acutatum* infection (Figure 5C) except for FaPR1-1, a well-known SA-pathway-associated gene. The same result was found in petiole (Figure S2C). Nearly all strawberry SA-associated transcripts including FaPR1-1, increased in abundance in response to SA treatment. Two classical SA-associated PR orthologous genes, FaPR1-2 and FaPR2-2, are shown to be mainly JA-dependent in strawberry (Figure 6C).

### Level of SA and JA during the strawberry/*C. acutatum* interaction

**Figure 7:**
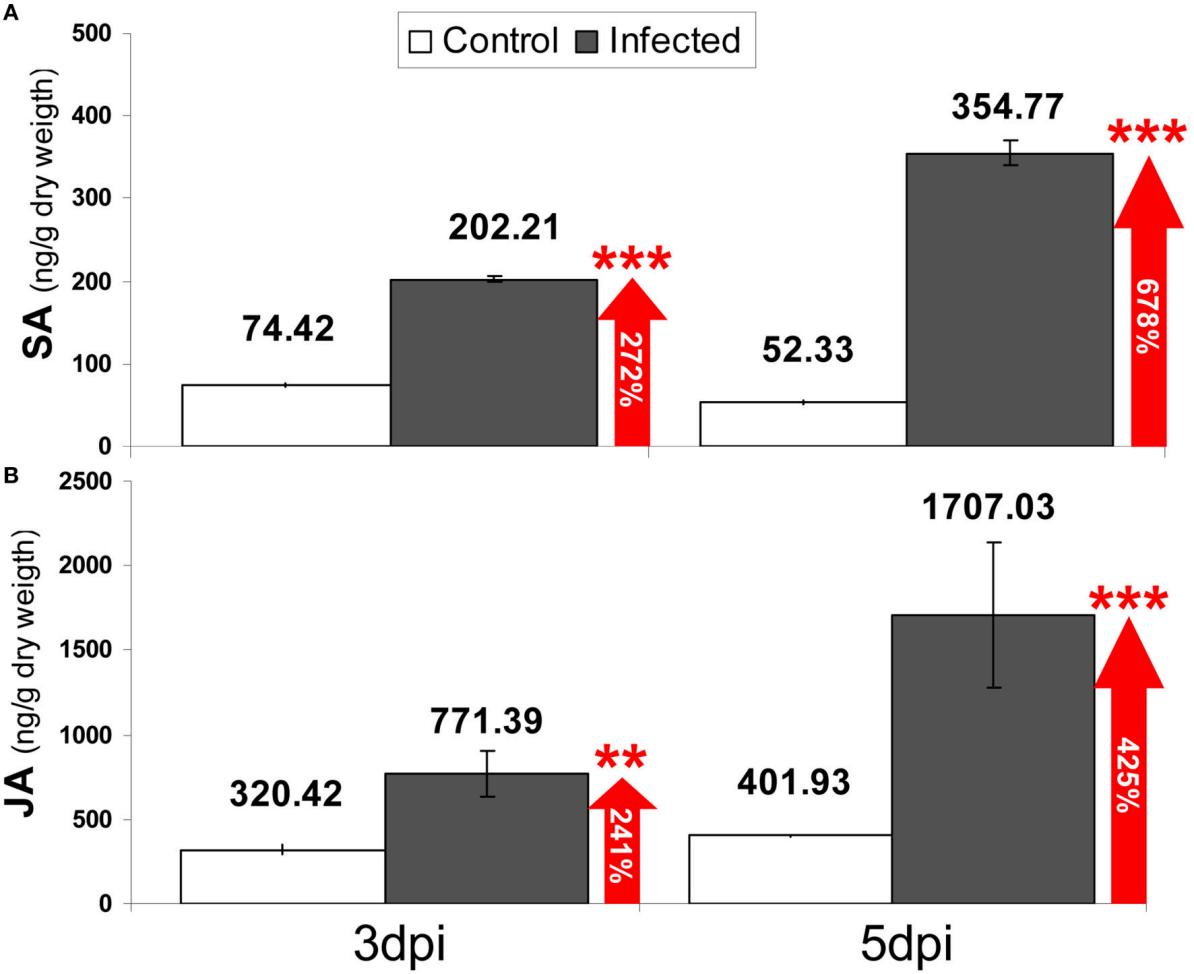
**Quantification of (A) SA and (B) JA [ng g-1 (dry weigh)] in aerial tissues of *in-vitro* strawberry plantlets at 3 and 5 days after mock (white bars) and pathogen inoculation (black bars)**. Data are the mean of three biological replicates, and error bars represent the SD. Numbers inside arrows indicate percentage of increase of infected samples vs. mock. Asterisks indicate confidence of ANOVA-Bonferroni Multiple Comparisons Test (^***^p < 0.001; ^**^p < 0.01).

Salicylic acid (SA) and jasmonic acid (JA) content in strawberry plantlets cv. Camarosa was measured at 3 and 5 days after inoculation with *C. acutatum*. A significant 272% increase in free SA concentration was detected in aerial tissues only at 3 dpi (202.21 ng/g dw, in infected plantlets vs. 74.42 ng/g dw, in mock-treated plantlets; Figure 7A). In addition, free SA in infected plantlets increased up to 678% at 5 dpi compared with that of mock treatment (354.77 ng/g dw vs. 52.33 ng/g dw, respectively). A significant 241% increase in free JA concentration was detected in infected plantlets at 3 dpi compared with that of mock treatment (771.39 ng/g dw, vs. 320.42 ng/g dw, respectively; Figure 7B). This increase was even higher after 5 dpi (425%), compared with that of mock treatment (1707.03 ng/g dw vs. 401.93 ng/g dw, respectively).

## Discussion

### Disease progress

This report characterizes the molecular events related to strawberry response to *C. acutatum* infection largely prior to plant disease symptom occurrence. In other pathosystems, main changes in gene expression are known to occur before significant lesion development (Windram et al., 2012). Previous research indicates that *C. acutatum* may persist and grow extensively on strawberry tissues without causing visible symptoms (Freeman et al., 1998; Leandro et al., 2001; Debode et al., 2009). Under inoculation conditions (10^4^ conidia·ml^−1^) used herein, no symptoms were visible during the time course of the experiment. Conditions mirror those used in previously published work (Casado-Díaz et al., 2006; Encinas-Villarejo et al., 2009; Amil-Ruiz et al., 2012, 2013). Transmission electron microscopy results (Figure 2) are roughly consistent with the timing of pathogen development observed after spray inoculation, taking into account that spot inoculation with a higher spore concentration (10^6^ conidia·ml^−1^) is forcing a much quicker infection process. Indeed, this inoculum concentration is also used by Horowitz et al. (2002) to study the developmental stages of this pathogen in strawberry, and necrotrophic transition was first identified after 4 dpi, which is consistent with our TEM observations. In addition, the penetration phase of *C. acutatum* on strawberry tissues is not a synchronous process, as previously described for this pathogen and other *Colletotrichum* spp. on other hosts (Makowski and Mortensen, 1998; Arroyo et al., 2005). Furthermore, at 5 days after inoculation, less than fifty percent of the conidia showed fully formed appresoria, and limited primary hyphae formation was observed (Figure 1). To strengthen the transcriptomic analysis, a more detailed gene expression study (RTqPCR) was performed throughout the *C. acutatum* infection stages from 1 to 9 days after inoculation, which certainly should reflect changes in host in response to most pathogen development stages. A similar time-window for gene expression has been monitored in other pathosystems (Doehlemann et al., 2008; Marcel et al., 2010; Vargas et al., 2012). Previous studies on *C. acutatum*-strawberry interaction have also shown that main transcript changes occur in host along the period of time herein analyzed (Casado-Díaz et al., 2006; Encinas-Villarejo et al., 2009). All in all, these observations informed about the appropriate time points for transcriptomic analyses.

### Molecular components of the strawberry response to *C. acutatum* identified in this study

Following *C. acutatum* infection, the transcriptomic analysis shows a wide range of responses to this pathogen. Many of the identified transcripts encode proteins with demonstrated roles in resistance and defense functions. Other transcript classes identified include members of plant pathogen perception and sensing apparatus, signal transduction machinery, transcriptional factors and regulatory genes, and protein synthesis and secretion mechanisms. For the purposes of this report, only components belonging to known SA and JA signaling pathways are discussed.

#### Signaling transduction pathways: Downstream responses against C. acutatum

In model plants, one of the big gaps in plant immunity understanding lies in the signaling pathways operating immediately downstream of PRR and R protein activation. However, partially understood pathways have been established (Dodds and Rathjen, 2010). We have found that members of kinase, phosphatase, ubiquitin and calcium-associated gene families related to signal transduction pathways were induced upon interaction with *C. acutatum* (Tables 1A,B and Table S3). Known components of both SA- and JA-dependent defense signaling pathways were also up-regulated. No induction was observed of important components of MAP Kinases pathway, which seems to play a role in the interplay between SA and JA-defense signaling pathways (Rodriguez et al., 2010; Caarls et al., 2015). The reasons behind this are unclear, but it is possible that the pathogen could be manipulating part of this pathway.

#### SA-signaling pathway

Enrichment of transcripts corresponding to specific SA-pathway members was detected. Thus, the expression of genes FaEDS1 (EDS1-936^EST^) and FaPAD4 (M4E10^EST^) is induced by *C. acutatum*. The lipase-like protein EDS1 is an important node acting upstream of SA in PAMP-triggered immunity (PTI) after PRRs stimulation and is also required for signaling of all TIR-NB-LRRs tested to date (Wiermer et al., 2005; Heidrich et al., 2011). These findings suggest that specific effector-triggered immunity (ETI) through TIR domain signaling might also be acting in strawberry against this pathogen. EDS1 is known to physically interact with two other positive regulators, PAD4 and SAG101, both of which are putative lipases, although hydrolase activity has not been demonstrated for either protein (Wiermer et al., 2005). Interestingly, the expression of Arabidopsis EDS1 is positively regulated by WRKY70 transcription factors (Li et al., 2004), and enrichment in WRKY70 orthologs has also been detected in strawberry (see further below). Moreover, a strawberry PAD4 ortholog (FaPAD4) was indeed upregulated. PAD4 affect SA accumulation (Wang et al., 2011). Thus, the dissociated forms of EDS1 and PAD4 are fully competent in signaling receptor triggered localized cell death at infection locations (Rustérucci et al., 2001; Aviv et al., 2002) but, by contrast, an EDS1–PAD4 complex is necessary for basal resistance involving transcriptional up-regulation of PAD4 itself and mobilization of salicylic acid defenses (Rietz et al., 2011).

Local SA production has been shown to trigger defenses in the surrounding cells downstream of EDS1 and PAD4 activity. This SAR is activated thorough a systemic signal that primes distal tissues against similar invaders. The SA derivative methyl salicylate (MeSA) is believed to serve as a long-distance phloem-mobile SAR signal in plants (Liu et al., 2011; Dempsey and Klessig, 2012). Once in the distal, uninfected tissue, MeSA must be converted into biologically active SA by esterase activity (Dempsey and Klessig, 2012). Interestingly, induction of the strawberry gene M9D5^EST^ encoding a methyl salicylate (MeSA) esterase similar to the Arabidopsis AtMES9 was detected (Tables 1A,B), which suggests that this signaling mechanism might also be activated in strawberry during *C. acutatum* interaction. Curiously, the Arabidopsis AtMES9 presents *in-vitro* activity with MeSA, MeJA and MeIAA (Yang et al., 2008) but it showed preference for MeSA as a substrate (Vlot et al., 2008; Dempsey and Klessig, 2012).

Induction of other transcripts acting downstream of SA was detected. Two WRKY70-like genes, FaWRKY70-1 (M17H1^EST)^ and FaWRKY70-2 (M12E12^EST^), and a glutaredoxin GRX480-like gene, FaGRX1 (M30F8^EST^), which have been described as essential components for SA-dependent defense activation, were detected in strawberry after infection. In addition, transcript accumulation of strawberry orthologs to classical SA associated genes such as PRs FaPR1-2, FaPR2-1, and FaPR2-2 was highly induced in crown or petiole (Figure 5C and Figure S2C). Recently the FaPR2-2 transcript has been reported as a reliable indicator of SA-dependent defenses in strawberry, as it was induced by *C. acutatum, C. fragariae*, and SA (Zamora et al., 2012). However, although FaPR1-2, FaPR2-1 and FaPR2-2 were detected in this study after SA treatment, both FaPR1-2 and FaPR2-2 turned out to be also induced after JA treatment (Figure 6C). Therefore, we propose that these two later genes should not be considered as SA-specific transcripts in strawberry.

*C. acutatum* infection also increased M8G7^EST^ transcript, encoding a protein that resembles the HSF-like transcription factor TBF1, a member of an extensive family of heat responsive proteins (Baniwal et al., 2004; Ikeda and Ohme-Takagi, 2009). These proteins are associated with diverse functions, including heat stress response (Charng et al., 2007; Ikeda et al., 2011), and plant development (Pernas et al., 2010; ten Hove et al., 2010). Interestingly, the TBF1 protein has recently been shown to be a major molecular switch for plant growth-to-defense transition in Arabidopsis (Pajerowska-Mukhtar et al., 2012). This transcription factor is a positive regulator of immune responses induced by salicylic acid and PAMPs, and it binds to the TL1 (GAAGAAGAA) *cis* element of NPR1-dependent ER-resident genes required for antimicrobial protein secretion.

#### JA-signaling pathway

Transcripts related to the JA-mediated signaling pathway were also induced in strawberry after *C. acutatum* infection, including FaAOS-1 (M28A2^EST^), FaWRKY33-1 (M8H3^EST^), and FaWRKY33-2 (M1C12^EST^). FaAOS-1 encodes an allene oxide synthase, a member of the cytochrome p450 CYP74 gene family (Song et al., 1993) that functions as a key enzyme in the initial steps of the JA biosynthetic pathway (Peña-Cortés et al., 2004; Leon-Reyes et al., 2010), thus generating signaling molecules that are essential for host immunity and plant development (Acosta and Farmer, 2010; Gfeller et al., 2010; Bak et al., 2011). Interestingly, while only a single copy of AOS gene exists in Arabidopsis (Kubigsteltig et al., 1999), a small AOS gene family with five members can be detected in *F. vesca* genome (unpublished), and three AOS members have been detected in tomato (López-Ráez et al., 2010), suggesting a more complex regulation of this pathway in fruiting plants. In addition, FaWRKY33-1 and FaWRKY33-2 are duplicated in *Fragaria* and can be paralogous, and both are similar to the well-known WRKY33 transcription factor from Arabidopsis. This important transcription factor acts downstream of JA and regulates expression of classical JA-dependent defense genes, such as those encoding glucanases, chitinases, and thaumatin-like proteins, which have been extensively described as JA-associated genes in other plants. Accordingly, many strawberry orthologs to these JA-induced proteins, such as FaGLN-2, FaCHI4-2, FaPR10-4, FaPR5-1, FaPR5-2, and FaPR5-3, were strongly induced by *C. acutatum* (Figure 5A).

Upregulation of genetic components needed for SA and JA synthesis in strawberry is accompanied by a concomitant increase in concentration of such phytohormones in response to inoculation by *C. acutatum* (Figure 7). Therefore, taken together, these results clearly demonstrate that both SA and JA defense signaling pathways are activated in strawberry during *C. acutatum* infection.

In addition, main transcript accumulation was observed between 3 and 5 dpi for genes *FaWRKY1, FaCHI4-2, FaPR10-4, FaPR5-2, FaPR5-3, FaLIP1, FaPR2-1*, and *FaPR2-2*, coding for known PR defense proteins (Figure 5). This increase roughly coincides with the break time observed during conidial germination and appressoria formation of *C. acutatum* (Figure 1), and it may reflect an early attempt of strawberry plant to halt pathogen development. After 5 dpi, expression of these genes decreases gradually while a progressive increase in the percentage of germinated conidia with fully developed appressoria occurs (Figure 1). The latter may reflect the process whereby pathogen hijacks plant defenses to its own benefit.

### Evidence of *C. acutatum* influence on SA- and JA-dependent defense pathways to promote pathogen development in strawberry

The SA and JA signaling pathways are activated in strawberry challenged with *C. acutatum*. However, results indicate that expression of well-known components of both SA- and JA-dependent defense pathways are not activated during this interaction. FaPR1-1 has been described as SA inducible in strawberry in cv. Pájaro, challenged with the avirulent strain M23 of *C. fragariae* but not after infection with virulent strain M11 of *C. acutatum* (Grellet-Bournonville et al., 2012). Infection with the avirulent strain M23 induced temporary SA accumulation (nearly 2-fold) in strawberry plants that was accompanied by induction of FaPR1-1 transcripts and protection against a subsequent infection with *C. acutatum*.

Here the infection of cv. Camarosa with *C. acutatum* induced the SA accumulation to a higher level (nearly 3-fold), but no significant accumulation of FaPR1-1 transcript above basal levels in crown and petiole was observed (Figure 5C and Figure S2C). In contrast, many other SA-responsive PRs (FaPR1-2, FaPR2-1, and FaPR2-1) were upregulated. The FaPR1-1 transcript did increase in abundance after SA treatment in “Camarosa,” which means that strawberry cv. Camarosa has the ability to induce this gene (Figure 6C).

No significant increase in FaGST1 expression (the strawberry AtGST1 heterolog, a well-known oxidative stress inducible transcript) was observed in crown and leaf tissue (Figures 3D,E) at any time after pathogen inoculation. Only a slight increase was observed in petioles after 5 dpi. These results suggest that little, if any, ROS production occurs during pathogen development. No significant DAB staining was evident in strawberry tissue during this time course, which is consistent with the results. It is known that ROS is generated in response to abiotic stress, particularly H_2_O_2_, which is an active signaling molecule triggering a variety of defense responses, with GST induction being the most significant (Bhattacharjee, 2012).

Production of H_2_O_2_ and activation of gene PR-1 gene occur simultaneously, and it has been suggested that H_2_O_2_ acts downstream from SA in the pathogenesis-related (PR-1) gene induction [73]. Intriguingly, no ROS production and no significant *FaGST* and *FaPR1.1* transcript induction were detected in strawberry after pathogen inoculation, even though SA increased. Our results strongly correlate with those of Grellet-Bournonville et al. (2012), who also reported that virulent *C. acutatum* strain M11 showed no ROS accumulation and no *FaPR1.1* gene induction after strawberry infection.

On the other hand, expression of strawberry orthologs to JA-associated defense related genes, such as FaPDF1, FaLOX2-1, and FaJAR1, also remained unchanged after infection with *C. acutatum*, despite the fact that many other components of the JA-mediated signaling pathway were induced (FaAOS-1, FaWRKY33-1, FaWRKY33-2; Figure 5B and Figure S2B).

Our results indicate that a number of known components of both SA- and JA-dependent defense pathways are not activated in strawberry during interaction with *C. acutatum*. Furthermore, our results support a hypothesis that *C. acutatum* might be interfering with certain branches of both SA- and JA-dependent defense pathways in strawberry. Absence of a significant plant defense response would allow successful colonization of host tissue by this pathogen. In this sense, recent results reported on the tomato-*Botrytis* system have shown that the exopolysaccharide production by this pathogen [EPS, known as b-(1,3)(1,6)-D-glucan] acted as elicitor of the tomato SA biosynthesis pathway and that inappropriate induction of SA by this pathogen impaired tomato JA-dependent defenses by interrupting the JA signaling pathway downstream of JA production (El Oirdi et al., 2011; Rahman et al., 2012). Consequently, the fungus could gradually spread through tomato plant tissues.

**Figure 8:**
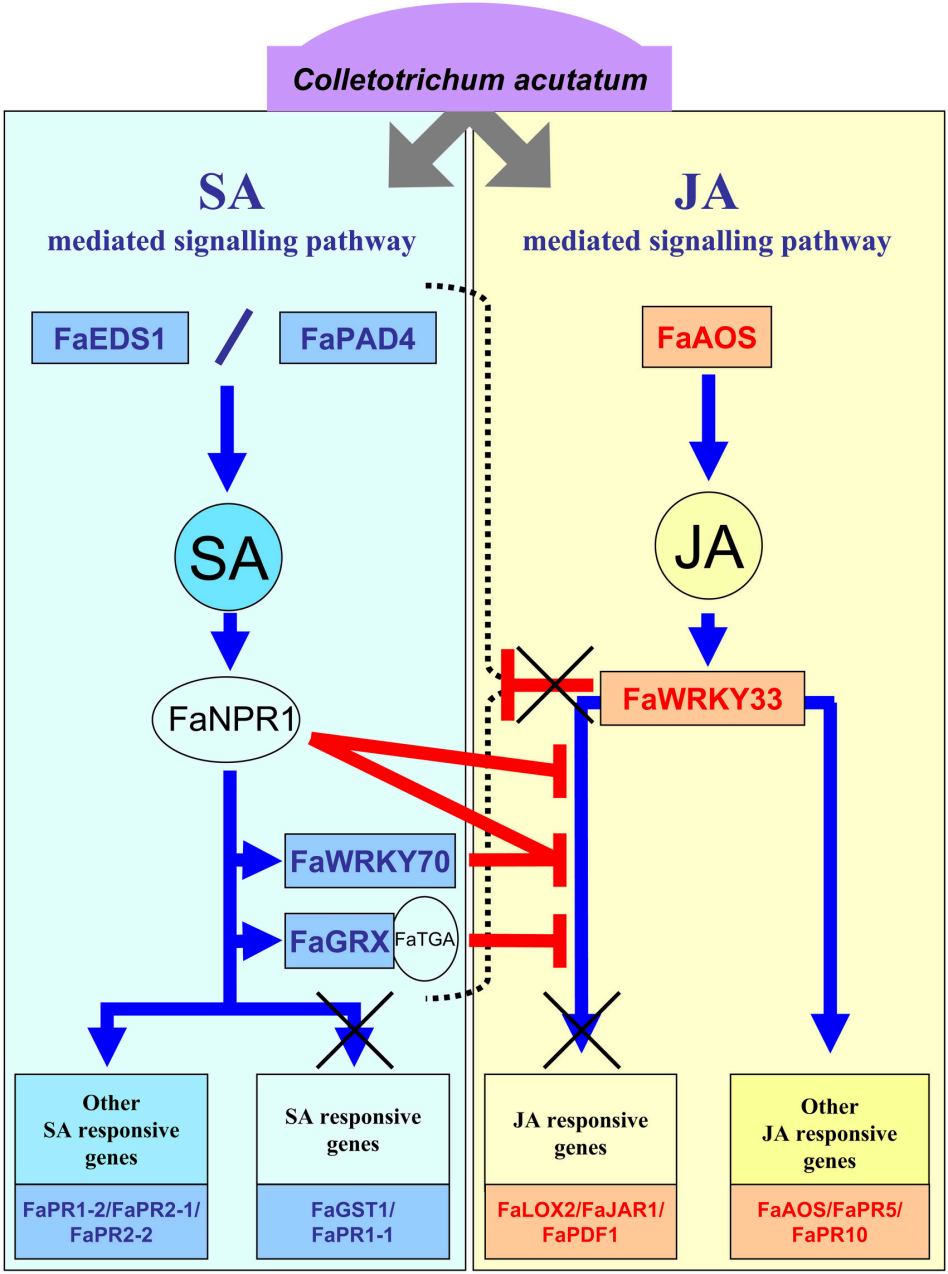
**Hypothetical model of SA- and JA-dependent defense pathways activated in strawberry in response to *C. acutatum***. This model is based on the canonical pathways described in model plant. Upon interaction with *C. acutatum*, the strawberry plant activates upstream components of SA and JA defense pathways. Thus, synthesis of these signal molecules increases and main downstream key components for SA (FaNPR1, FaWRKY70, FaGRX) and JA (FaWRKY33) are activated. Unlike Arabidopsis WRKY33, FaWRKY33 does not act as a negative regulator of the entire SA-dependent defense signaling pathway, either by a direct or an indirect effect of fungal activity, but only for some components (FaGST1, FaPR1.1). That allows FaGRX, together with FaNPR1 and FaWRKY70, to act as negative regulators of JA responsive genes similarly to their Arabidopsis orthologs. As a result, important JA-responsive defense marker genes, such as FaLOX2, FaJAR1, and FaPDF1, are not induced. These impaired mechanisms might provide some advantage for fungal spreading.

It is tempting to speculate that *C. acutatum* may be able to interact with strawberry defense response. Based on our results, and considering the canonical SA JA crosstalk model proposed in Arabidopsis (Spoel et al., 2007; Spoel and Dong, 2012), an integrated model has been devised (Figure 8). In this model, in which activation of both SA and JA pathways and increased amount of SA and JA signal molecules occurs after *C. acutatum* infection, negative crosstalk between these two pathways should somehow be expected. Spoel et al. (2007) showed that simultaneous *A. thaliana* inoculation with a biotrophic and a necrotrophic pathogen resulted in impaired resistance to the necrotrophic pathogen, and demonstrated that the SA pathway that was activated by the biotrophus suppressed the level of JA-dependent resistance against the necrotroph. Indeed, SA-mediated suppression of JA-responsive gene expression has been reported to be targeted downstream of JA biosynthesis (Leon-Reyes et al., 2010). Thus, GRX480 is an NPR1 dependent-SA-inducible class III glutaredoxin (Rouhier et al., 2006; Krinke et al., 2007) specific to land plants (Ziemann et al., 2009), which interacts with TGA factors and suppresses JA-responsive PDF1.2 transcription (Ndamukong et al., 2007; Zander et al., 2011). In addition, NPR1 and WRKY70 also acts downstream of the SA molecule as a node of convergence for JA-mediated and SA-mediated signals (Dong, 2004; Li et al., 2004; Wang et al., 2006; Ren et al., 2008), balancing the JA- and SA-dependent responses (Li et al., 2006). Interestingly, strawberry orthologs FaGRX1, FaWRKY70-1 and FaWRKY70-2 were specifically induced during interaction with *C. acutatum*, but no induction on FaPDF1 and other major components of JA-dependent signaling pathway, such as genes FaLOX2-1 and FaJAR1, was detected. Moreover, an increase in JA synthesis and upregulation of FaAOS-1, the strawberry ortholog to Arabidopsis AtAOS, a well-known JA-associated marker gene encoding a key enzyme for JA synthesis, was also reported after *C. acutatum* infection, supporting the idea that in strawberry repression of some JA-responsive genes is targeted downstream of the JA biosynthesis. Indeed, AtAOS has been described as a MeJA-inducible gene but not suppressed by WRKY70 (Li et al., 2006). Very interestingly, the expression of a second group of known JA-responsive genes, such as FaGLN-2, FaCHI4-2, FaPR10-4, FaPR5-1, FaPR5-2, FaPR5-3, increased after being challenged with this pathogen, suggesting the presence in strawberry of a second GRX480/WRKY70-independent JA-dependent defense branch.

Importantly, two other JA-associated AtWRKY33-like genes, FaWRKY33-1 and FaWRKY33-2, were also upregulated in strawberry by *C. acutatum*. The JA-associated component AtWRKY33 has recently been reported as a key transcriptional regulator of defense responses to necrotrophus (Birkenbihl et al., 2012). Indeed, AtWRKY33 acts as a negative regulator of the SA-defense pathway upon pathogen infection and negatively controls expression of many important genes, including those responsible for SA biosynthesis and accumulation, positive regulatory proteins EDS1 and PAD4, and SA responsive genes PR1, PR2, and PR3. Interestingly, in strawberry, expression of the SA-dependent orthologous genes FaGST and FaPR1-1 remained unaltered but very intriguingly, the synthesis of SA and the expression of orthologs to components of SA-mediated signaling pathway acting upstream (FaEDS1 and FaPAD4), and downstream of SA (FaGRX1, FaWRKY70-1, FaWRKY70-2, FaPR1-2, FaPR2-1, and FaPR2-2), was remarkably induced during the infection with *C. acutatum*, despite the fact that FaWRKY33-1 and FaWRKY33-2 were clearly upregulated. Unlike what has been previously described for AtWRKY33 (Birkenbihl et al., 2012), these results suggest that repressive control of many known components of the SA-pathway through these FaWRKY33 transcription factors does not work in strawberry during interaction with *C. acutatum* (Figure 8).

In conclusion, our results demonstrate both that SA and JA increase in strawberry upon *C. acutatum* infection, and that known plant defenses through SA and JA dependent signaling pathways are partially promoted during such interaction. Indeed, major plant defense marker genes are not up-regulated in strawberry after infection with this pathogen, which might evidence a putative molecular strategy used by this pathogen to overcome strawberry plant defense. This is in line with the new emerging paradigm that a key pathogen virulence strategy involves modulation of plant hormone signaling (El Oirdi et al., 2011; Chung et al., 2013).

Results from our research are of great use to further our understanding of the strawberry immune system to enable future disease control through biotechnological and breeding strategies.

## Author contributions

FAR and supervisors JC and JMB conceived and designed the experiments. FAR, JC, and JMB contributed to reagents/materials and analysis tools. FAR, JGG, JG, RB, AM, OT, BD, FR, JAM, FP carried out experiments, analyzed and interpreted the data. FAR, JC contributed to drafting the manuscript. AA, FA, JGG, FAR, JC performed the light and TEM microscopic observations. JC, JMB, JG revising the article critically. All authors approved the final version of the manuscript.

### Conflict of interest statement

The authors declare that the research was conducted in the absence of any commercial or financial relationships that could be construed as a potential conflict of interest.

## Abbreviations

EPS: exopolysaccharide
ER-resident: endoplasmic reticulum-resident
ET: ethylene
ETI: effector-triggered immunity
FDR: false discovery rate
JA: jasmonic acid
PAMP: pathogen-associated molecular pattern
PR proteins: pathogenesis related proteins
PRR: pattern recognition receptor
PTI: PAMP-triggered immunity
qPCR: quantitative PCR
R protein: resistance protein
SA: salicylic acid
SEA: singular enrichment analysis
