# Supplementary material for: Partial Activation of SA- and JA-Defensive Pathways in Strawberry upon Colletotrichum acutatum Interaction
Source: Front Plant Sci. 2016 Jul 15;7:1036. doi: 10.3389/fpls.2016.01036 (PMC4945649; doi:10.3389/fpls.2016.01036)
Supplement: Supplementary file 2 [file Table2.PDF]

Table S2. Identification of *F. vesca* new orthologous genes. Mostly, all ESTs matched with *F. vesca* predicted genes with a range of sequence identity between 95-99%. Thirteen *F* × *ananassa* ESTs did not match within the coding region of *F. vesca* predicted genes, and represented sequences either from the UTR regions of such genes or from not predicted ones. These *F* × *ananassa* sequences were blasted against the *F. vesca* pseudomolecules data, which represent all the strawberry genomic sequences linked into putative chromosomes, so that it allowed the identification of a specific fragment of genomic sequence, and the search within it for the exact location of the EST of interest. Thus, ESTs M5B6, M27C10, M23C7, M16H1, M25G5, M22G7, M30F8, M4F8, M22E11 and M22D5 represented 3´UTR regions, and EST M28B7 represented 5´UTR regions, both from predicted *F. vesca* genes. These genes are named in Table I as *F. vesca* orthologs “genexxxxx.3utr”, and “genexxxxx.5utr”, respectively. The M19D11 EST was found to be part of a putative intron within the corresponding *F. vesca* predicted gene. However, comparisons with Arabidopsis and other plant orthologs showed that this EST sequence was indeed part of an exon region encoding a kinase domain of the corresponding predicted protein. Similarly, the M12B6 EST was found to be part of a predicted 4kb long *F. vesca* intergenic region but blastx comparisons of this 4kb intergenic sequence against UniREF and TAIR10pro database (http://www.arabidopsis.org/) allowed us to predict a gene encoding a DNA-directed RNA polymerase TFIIB zinc-binding protein. The same methodology was used to identify other *F. vesca* orthologous genes and to assign putative functional roles to other *F* × *ananassa* ESTs sequences. In addition, six *F. vesca* gene predictions (gene19270, gene13677, gene05017, gene06367, gene12874, gene25662) were found to contain putatively more than one unique CDS, as their translated products matched different genes in the transcriptome of Arabidopsis and other species. For instance, the M2F10-orthologous *F. vesca*-predicted gene19270 sequence turned out to contain two putative CDS (we have named *F. vesca* gene19270.A and gene19270.B) when it was compared with the Arabidopsis proteome (the consecutive genes AT4G00340 and AT4G00350). Similar results were obtained when it was compared with other species at UniRef. A distance of approximately 1.5kb exist between these two new *F. vesca* predicted CDS, which expands over the *F. vesca* predicted gene19270 sequence. However, this *F. vesca* genomic region had been predicted as an intron expanding from within the 3´UTR region of the first new gene to within the promoter region of the next one. Therefore, *F* × *ananassa* M2F10^{EST} sequence matched part of the last exon and the 3´UTR region including the poly-A tail signal of the first one of these two new *F. vesca* predicted orthologous genes (*F. vesca* gene19270.A). Then, a putative functional *Receptor-like protein kinase 4* role was assigned to its encoded product according to its corresponding *A. thaliana* ortholog (AT4G00340). Altogether, these new predicted *F. vesca* genes should be appropriately annotated within future reports on *F. vesca* genome studies.

**FaARRAY ID**	***F. vesca* Ortologe**	**Position of the *F x ananassa* EST with respect to the predicted *F. vesca* orthologe gene CDS or fail in *F. vesca* gene architecture prediction.**
M5B6	gene24296	Part of the 3´utr. EST starts aproximately 5-8bp from the predicted stop codon. Next predicted gene ATG is located over 800bp from the final of the EST.
M27C10	gene30942	Part of the 3´utr. EST starts aproximately 35bp from the predicted stop codon. Next predicted gene ATG is located over 900bp from the final of the EST.
M23C7	gene25539	Part of the 3´utr. EST starts aproximately 50bp from the predicted stop codon. Next predicted gene ATG is located over 1800bp from the final of the EST.
M16H1	gene14094	Part of the 3´utr. EST starts aproximately 260bp from the predicted stop codon. Next predicted gene ATG is located over 1500bp from the final of the EST.
M25G5	gene06563	Part of the 3´utr. EST starts aproximately10bp before the predicted stop codon. Next predicted gene stop codon is located over 900bp from the final of the EST (oriented in negative frame).
M22G7	gene09933	Part of the 3´utr. EST starts aproximately 30bp from the predicted stop codon. None gene is predicted over 1000bp from the final of the EST.
M30F8	gene29769	Part of the 3´utr. EST starts aproximately 25bp from the predicted stop codon. None gene is predicted over 1000bp from the final of the EST.
M4F8	gene15022	Part of the 3´utr. EST starts aproximately 50bp from the predicted stop codon. None gene is predicted over 1000bp from the final of the EST.
M28B7	gene16235	Part of the 5´utr. EST ends aproximately 300bp upstream of the predicted ATG. None gene is predicted 1000bp upstream from the begining of the EST.
M22E11	gene15974	Part of the 3´utr. EST starts aproximately 400bp after the predicted stop codon. Next gene stop codon is located over 1800bp from the final of the EST (oriented in negative frame).
M22D5	gene31183	Part of the 3´utr. EST starts aproximately 600bp after the predicted stop codon. Next gene ATG is located over 3000bp from the final of the EST.
M19D11	gene10418	The EST is part of one intron with aproximately 700bp in lengh in the *F. vesca* predicted gene, but investigation on gene architecture of Arabidopsis and other plant orthologes shows that with high provability that region is really an exon instead of an intron, codifying for part of a kinase domain, and so, prediction of *F. vesca* gene could be failed in the detection of intron-exon signals, for this particular case.

M12B6	no hit found	There is no gene predicted in 4kb arround the position of the EST in *F. vesca* genome, but searching for putative CDS in that 4kb fragment, by blastx against UniRef and TAIR10pro, shows that contains a gene annotated as *DNA-directed RNA polymerase TFIIB zinc-binding protein*, but was failed to be predicted in *F. vesca* transcriptome*. F x ananassa s*equence is part of the first exon of this unpredicted gene.
M2F10	gene19270	Clear fail in *F. vesca* prediction of gene (gene19270) which contain two putative CDS (in Arabidopsis genome are consecutive genes), and the F x ananassa associated sequence (M2F10) represent part of the last exon and 3´utr zone of the first of both genes, containing even the poly-A tail. Error in prediction consist on prediction of intergene region of almost 1,5kb as intron.
M27A2	gene13677	Fail in *F. vesca* prediction of gene (gene13677) which contain two putative CDS (in Arabidopsis genome are consecutive genes), and the F x ananassa associated sequence (M27A2) represent part of the first exon of the second of both genes. Error may consist on prediction of intergene region of almost 3kb as intron.
M4E4	gene05017	Fail in *F. vesca* prediction of gene (gene05017) which contain two putative CDS (in Arabidopsis genome are located in different cromosomes), and the *F x ananassa* associated sequence (M4E4) represent part of the last exon and 3´utr zone of the first of both genes, containing even the poly-A tail. Error consist on prediction of intergene region of almost 1,5kb as intron.
M17E3	gene06367	Fail in F. vesca prediction of gene (gene06367) which contain two putative CDS, and the F x ananassa associated sequence (M17E3) represent part of the first exon of the second of both genes. Error may consist on prediction of intergene region of almost 1kb as intron.
M1H8	gene12874	Fail in F. vesca prediction of gene (gene12874) which contain three putative CDS, and the F x ananassa associated sequence (M1H8) represent part of the first exon of the third of those genes. Error may consist on prediction of two intergene regions of almost 1,5kb and 0,8kb as introns.
M28F7	gene25662	Fail in F. vesca prediction of gene (gene25662) which contain two putative CDS, and the F x ananassa associated sequence (M28F7) represent aproximately 40bp of the 5´utr and part of the first exon of the second of both genes. Error consist on predict intergene region of almost 1,5kb as intron.
