# Supplementary material for: Partial Activation of SA- and JA-Defensive Pathways in Strawberry upon Colletotrichum acutatum Interaction
Source: Front Plant Sci. 2016 Jul 15;7:1036. doi: 10.3389/fpls.2016.01036 (PMC4945649; doi:10.3389/fpls.2016.01036)
Supplement: Supplementary file 3 [file Table3.PDF]

Table S3a. Up-regulated genes with predicted role in defence response against *C. acutatum* and associated references. A selection of references linking these genes to defence response in plants is showed. Color code of each group of genes is associated with functional classification as in Table I.

Strawberry gene ID	*A. thaliana* Orthologue	Relation with Defense/Function	References
**Invasion sensing**			
M14D5	AT1G30240	LRR protein, plant receptor	Ascencio-Ibáñez *et al.*, 2008; Kiba *et al.*, 2005
M6C2	AT5G42090	Plant receptor	Dunkley *et al.*, 2004; Ascencio-Ibáñez *et al.*, 2008; Moriyama *et al.*, 2006
ELRR-39	AT5G21090	LRR protein, plant receptor	Tamaoki *et al.*, 2003; Li *et al.*, 2006; Ascencio-Ibáñez *et al.*, 2008; Schenk *et al.*, 2003; Gou *et al.*, 2009
M29F3	AT3G14460	CC-NBS-LRR class of R proteins, plant receptor	Meyers *et al.*, 2003; Tan *et al.*, 2007; Cannon *et al.*, 2002; Ashfield *et al.*, 2004
M18E3	AT3G14460	CC-NBS-LRR class of R proteins, plant receptor	Meyers *et al.*, 2003; Tan *et al.*, 2007; Cannon *et al.*, 2002; Ashfield *et al.*, 2004
M13C5	AT5G13160	Receptor kinase, R protein-guard model	Swiderski and Innes, 2001; Shao *et al.*, 2003; DeYoung *et al.*, 2012; Day and He, 2010; Takemoto *et al.*, 2011; Zhang *et al.*, 2010; Caldwell and Michelmore, 2009
M19F7	AT4G33210	Fbox/LRR protein, plant receptor, Proteasome complex	Xiao and Jang, 2000; McKinney *et al.*, 2002; Callis and Vierstra, 2000; Lohmann *et al.*, 2010
M2F10	AT4G00340	Receptor kinase, Signal transduction regulation	Samuel *et al.*, 2008; Du and Chen, 2000
**Signal Transduction**			
M13C5	AT5G13160	Receptor kinase, R protein-guard model	Swiderski and Innes, 2001; Shao *et al.*, 2003; DeYoung *et al.*, 2012; Day and He, 2010; Takemoto *et al.*, 2011; Zhang *et al.*, 2010; Caldwell and Michelmore, 2009
M19F7	AT4G33210	Fbox/LRR protein, plant receptor, Proteasome complex	Xiao and Jang, 2000; McKinney *et al.*, 2002; Callis and Vierstra, 2000; Lohmann *et al.*, 2010
M2F10	AT4G00340	Receptor kinase, Signal transduction regulation	Samuel *et al.*, 2008; Du and Chen, 2000
M23A9	AT4G35790	Phospholipase D, Transduction of stress responses	Katagiri *et al.*, 2001; McGee *et al.*, 2003; Zhang *et al.*, 2003; Uraji *et al.*, 2012
M27D3	AT5G01160	E3 ligase, Proteasome complex	Delauré *et al.*, 2008

M16B7	AT1G69960	Ser/Thr protein phosphatase 2A catalytic subunit	Bíró *et al.*, 2012; Trotta *et al.*, 2011b; Trotta *et al.*, 2011a
M19D11	AT3G03940	Calcium binding kinase	Hrabak *et al.*, 2003; Kolukisaoglu *et al.*, 2004; Ferrando *et al.*, 2001; Xie *et al.*, 2010
M23A6	AT4G11740	Ubiquitin, Proteasome complex	Delauré *et al.*, 2008
M27C10	AT5G25510	Ser/Thr protein phosphatase 2A catalytic subunit	Bíró *et al.*, 2012; Trotta *et al.*, 2011b; Trotta *et al.*, 2011a
M8G2	AT4G30960	Calcium binding kinase SOS2	Hrabak *et al.*, 2003; Kolukisaoglu *et al.*, 2004; Ferrando *et al.*, 2001; Xie *et al.*, 2010; Fabro *et al.*, 2008
M4F10	AT1G65430	E3 ligase, Proteasome complex	Delauré *et al.*, 2008; Mladek *et al.*, 2003; Stone *et al.*, 2005; Kraft *et al.*, 2005
M8G7	AT4G36990	Major molecular switch for plant growth-to-defense transition	Pajerowska-Mukhtar *et al.*, 2012
M10E2	AT1G27460	Calmoduling binding protein	Brodersen *et al.*, 2006
M3D5	AT1G05180	Fbox, JA signaling, Proteasome complex	del Pozo *et al.*, 2002; Xu *et al.*, 2002; Lorenzo and Solano, 2005; Dharmasiri *et al.*, 2007; Hotton *et al.*, 2011; Merlet *et al.*, 2009
M25E7	AT1G15780	Interact with calciun binding protein kinase	Thimm *et al.*, 2004
M13H9	AT5G57020	Co-traslational addition of myristic acid	Qi *et al.*, 2000; Boisson *et al.*, 2003
M7G11	AT1G69640	Synthesis of membrane components	Peer *et al.*, 2010
M4C3	AT5G10930	Calcium binding kinase	Hrabak *et al.*, 2003; Kolukisaoglu *et al.*, 2004; Ferrando *et al.*, 2001; Xie *et al.*, 2010
M14H1	AT3G51860	Proton/Calcium antiporter	Cheng *et al.*, 2004; Cheng *et al.*, 2005; Barkla *et al.*, 2008; Manohar *et al.*, 2011
M7B6	AT1G80210	Homologous recombination, Deubiquitinating activity, Proteasome complex	Block-Schmidt *et al.*, 2011
M21H5	AT5G56180	Fbox/Actin/helicase domain, Proteasome complex, XXXRNAmetabolism	
M4E6	AT4G33240	Protein kinase, Protein trafficking, Endomembrane homeostasis	Hirano *et al.*, 2011; Hirano and Sato, 2011
M17E3	AT4G24830	NO synthesis, Signal transduction	Tischner *et al.*, 2007; Besson-Bard *et al.*, 2008; Wilson *et al.*, 2008
M10B6	AT3G13460	Calcium transport to nucleus, regulate gene expression	Ok *et al.*, 2005
M13F3	AT3G27925	Protease	Chassin *et al.*, 2002; Sinvany-Villalobo *et al.*, 2004; Zienkiewicz *et al.*, 2012
M1H8	AT5G53360	E3 ligase, Proteasome complex	Delauré *et al.*, 2008; Zhang *et al.*, 2007; Ryu *et al.*, 2010

M8D11	AT1G60490	Protein kinase, Protein trafficking, Secretory Pathway	Lee *et al.*, 2008; Leshem *et al.*, 2007; Whitley *et al.*, 2009; Wang *et al.*, 2012; Wang *et al.*, 2005; Kwon *et al.*, 2008; Wang and Dong, 2011; Pajerowska-Mukhtar *et al.*, 2012
M24D7	AT5G40150	Class III peroxidase	Andersson-Gunnerås *et al.*, 2006
M28C8	AT1G05260	Class III peroxidase	Andersson-Gunnerås *et al.*, 2006; Kim *et al.*, 2010
M3E6	AT1G71695	Class III peroxidase	Andersson-Gunnerås *et al.*, 2006; Schenk *et al.*, 2003
M4E10	AT3G52430	Lipase, Chemical defenses, SA pathway regulator	Rustérucci *et al.*, 2001; Rietz *et al.*, 2011; Louis *et al.*, 2010a; Louis *et al.*, 2012
EDS1-936	AT3G48090	Lipase, SA pathway regulator	Parker *et al.*, 1996; Heidrich *et al.*, 2011; Bhattacharjee *et al.*, 2011; Rietz *et al.*, 2011
**New Protein Synthesis and Secretion**			
M8D11	AT1G60490	Protein kinase, Protein trafficking, Secretory Pathway	Lee *et al.*, 2008; Leshem *et al.*, 2007; Whitley *et al.*, 2009; Wang *et al.*, 2012; Wang *et al.*, 2005; Kwon *et al.*, 2008; Wang and Dong, 2011; Pajerowska-Mukhtar *et al.*, 2012
M21B3	AT5G13080	Transcription factor	
M8H8	AT4G17960	RNA metabolism	Li *et al.*, 2008; Linder and Owttrim, 2009
M26G7	AT2G25970	RNA metabolism	Peal *et al.*, 2011; De Paepe *et al.*, 2004; Pavet *et al.*, 2005; Lorković *et al.*, 2000
M22D9	AT3G51980	Protein folding	Kamauchi *et al.*, 2005
J_4-9	AT5G13080	Transcription factor	Encinas-Villarejo *et al.*, 2009
M11C6	AT1G69620	Protein synthesis	Baima *et al.*, 1995; Barakat *et al.*, 2001; Carroll *et al.*, 2008
M6G7	AT3G48030	Transcription factor	Stone *et al.*, 2005; Kosarev *et al.*, 2002; Gracey *et al.*, 2001
M10C12	AT1G75780	Citosqueleton	Snustad *et al.*, 1992; Vassileva *et al.*, 2005; Wang and Dong, 2011; Lee *et al.*, 2012
M9F6	AT1G28420	Transcription factor	Son *et al.*, 2004
M1A2	AT1G62020	Protein transport	Bassham *et al.*, 2008
M23C4	AT4G37750	Transcription factor	Alvarez-Buylla *et al.*, 2010; Losa *et al.*, 2010; Holst *et al.*, 2011; Krizek and Eaddy, 2012; Krizek, 2011; Smith and Long, 2010; Krizek *et al.*, 2000
M18A9	AT5G46190	RNA metabolism	Peal *et al.*, 2011; De Paepe *et al.*, 2004; Pavet *et al.*, 2005; Lorković *et al.*, 2000
M7G4	AT3G52250	RNA metabolism	Schwartz *et al.*, 1994
M23C7	AT4G33865	Protein synthesis	Baima *et al.*, 1995; Barakat *et al.*, 2001; Carroll *et al.*, 2008

M18F1	AT1G47490	RNA metabolism	Peal *et al.*, 2011; De Paepe *et al.*, 2004; Pavet *et al.*, 2005; Lorković *et al.*, 2000
M11H4	AT3G12110	Citosqueleton	Huang *et al.*, 1997; Shimada *et al.*, 2006
M14B5	AT1G59740	Protein secretion	Chiang *et al.*, 2004
M5B8	AT5G22950	Protein secretion	Winter and Hauser, 2006
M3A1	AT3G16060	Citosqueleton	Lee and Liu, 2004; Lu *et al.*, 2005; Wei *et al.*, 2009
M19E4	AT2G44710	RNA metabolism	Peal *et al.*, 2011; De Paepe *et al.*, 2004; Pavet *et al.*, 2005; Lorković *et al.*, 2000
M18C5	AT1G66140	Transcription factor	Tague and Goodman, 1995; Englbrecht *et al.*, 2004; Schenk *et al.*, 2003
M3E11	AT1G18650	Cell-to-cell trafficking	Borner *et al.*, 2002; Borner *et al.*, 2003; Simpson *et al.*, 2009
M12B6	AT3G25940	RNA metabolism	Bäckström *et al.*, 2007
M7D1	AT3G05590	Protein synthesis	Baima *et al.*, 1995; Barakat *et al.*, 2001; Carroll *et al.*, 2008
M20A3	AT5G16715	Protein synthesis	Zhang and Somerville, 1997; Duchêne *et al.*, 2005
M8A6	AT1G77030	RNA metabolism	Li *et al.*, 2008; Linder and Owttrim, 2009
M9E2	AT1G80070	RNA metabolism	Schwartz *et al.*, 1994
M28B7	AT2G22430	Transcription factor	Söderman *et al.*, 1994; Himmelbach *et al.*, 2002
M6A9	AT5G67300	Transcription factor	Kranz *et al.*, 1998; Yanhui *et al.*, 2006; Libault *et al.*, 2007
M4C6	AT3G62310	RNA metabolism	Li *et al.*, 2008; Linder and Owttrim, 2009
M17H1	AT3G56400	Transcription factor, SA-JA crosstalk	Libault *et al.*, 2007; AbuQamar *et al.*, 2006; Hu *et al.*, 2012; Knoth *et al.*, 2007; Dong, 2004; Li *et al.*, 2004; Wang *et al.*, 2006; Ren *et al.*, 2008; Li *et al.*, 2006; Knoth *et al.*, 2007; von Saint Paul *et al.*, 2011
M8H3	AT2G38470	Transcription factor, JA pathway	Wan *et al.*, 2004; Libault *et al.*, 2007; Lippok *et al.*, 2007; Sarowar *et al.*, 2011; Birkenbihl *et al.*, 2012
M12E12	AT3G56400	Transcription factor, SA-JA crosstalk	Libault *et al.*, 2007; AbuQamar *et al.*, 2006; Hu *et al.*, 2012; Knoth *et al.*, 2007; Dong, 2004; Li *et al.*, 2004; Wang *et al.*, 2006; Ren *et al.*, 2008; Li *et al.*, 2006; Knoth *et al.*, 2007; von Saint Paul *et al.*, 2011
M1C12	AT2G38470	Transcription factor, JA pathway	Wan *et al.*, 2004; Libault *et al.*, 2007; Lippok *et al.*, 2007; Sarowar *et al.*, 2011; Birkenbihl *et al.*, 2012
**Direct Defences**			
M24D7	AT5G40150	Class III peroxidase	Andersson-Gunnerås *et al.*, 2006
M28C8	AT1G05260	Class III peroxidase	Andersson-Gunnerås *et al.*, 2006; Kim *et al.*, 2010
M3E6	AT1G71695	Class III peroxidase	Andersson-Gunnerås *et al.*, 2006; Schenk *et al.*, 2003

M4E10	AT3G52430	Lipase, Chemical defenses, SA pathway regulator	Rustérucci *et al.*, 2001; Rietz *et al.*, 2011; Louis *et al.*, 2010a; Louis *et al.*, 2012
M24B7	AT4G16260	Cell wall degradation, PR protein family	Shi *et al.*, 2006; Khan *et al.*, 2003; Ko *et al.*, 2007
M16D12	AT3G54420	PR protein family	Amil-Ruiz *et al.*, 2011; Takenaka *et al.*, 2009
EPR5-77	AT4G11650	PR protein family	Casado-Díaz *et al.*, 2006
M5B6	AT5G09360	Lignin biosynthesis	Pourcel *et al.*, 2005
M23A10	AT1G24020	PR protein family	Kim, 2011
M12C12	AT5G14180	Lipase, Chemical defenses	Yazaki *et al.*, 2004; Louis *et al.*, 2010b; Joe Louis, 2011
M6G11	AT4G34135	Secondary metabolism	Thilmony *et al.*, 2006; Langlois-Meurinne *et al.*, 2005
M6B9	AT1G24020	PR protein family	Kim, 2011
EPR5-284	AT4G11650	PR protein family	Casado-Díaz *et al.*, 2006
M1F10	AT1G20030	PR protein family	Osorio *et al.*, 2008
M22A10	AT1G24020	PR protein family	Kim, 2011
M5G8	AT1G24020	PR protein family	Kim, 2011
M10C5	AT1G24020	PR protein family	Kim, 2011
M26E5	AT5G17000	Redox protection	Mano *et al.*, 2005; Chivasa *et al.*, 2006; Zheng *et al.*, 2006
M4F3	AT1G22750	Secondary metabolism	Lasserre *et al.*, 2008; Yonekura-Sakakibara *et al.*, 2008
M25D10	AT1G24020	PR protein family	Kim, 2011
M5C8	AT4G32320	Antioxidant defences	Chew *et al.*, 2003
M23D11	AT4G37990	Lignin biosynthesis	Blanco-Portales *et al.*, 2002
M29A9	AT3G54420	PR protein family	Takenaka *et al.*, 2009
M25D11	AT3G07320	Cell wall degradation, PR protein family	Ko *et al.*, 2007
M10D7	AT1G24020	PR protein family	Kim, 2011
M26G2	AT2G30370	Inhibite stomatal production	Abrash and Bergmann, 2010; Shimada *et al.*, 2011
M21G5	AT1G69530	Stomatal movement	Zhang *et al.*, 2011; Wei *et al.*, 2011
**Hormone-Dependent Pathways**			
M4E10	AT3G52430	Lipase, Chemical defenses, SA pathway regulator	Rustérucci *et al.*, 2001; Rietz *et al.*, 2011; Louis *et al.*, 2010a; Louis *et al.*, 2012
EDS1-936	AT3G48090	Lipase, SA pathway regulator	Parker *et al.*, 1996; Heidrich *et al.*, 2011; Bhattacharjee *et al.*, 2011; Rietz *et al.*, 2011

M17H1	AT3G56400	Transcription factor, SA-JA crosstalk	Libault *et al.*, 2007; AbuQamar *et al.*, 2006; Hu *et al.*, 2012; Knoth *et al.*, 2007; Dong, 2004; Li *et al.*, 2004; Wang *et al.*, 2006; Ren *et al.*, 2008; Li *et al.*, 2006; Knoth *et al.*, 2007; von Saint Paul *et al.*, 2011
M8H3	AT2G38470	Transcription factor, JA pathway	Wan *et al.*, 2004; Libault *et al.*, 2007; Lippok *et al.*, 2007; Sarowar *et al.*, 2011; Birkenbihl *et al.*, 2012
M12E12	AT3G56400	Transcription factor, SA-JA crosstalk	Libault *et al.*, 2007; AbuQamar *et al.*, 2006; Hu *et al.*, 2012; Knoth *et al.*, 2007; Dong, 2004; Li *et al.*, 2004; Wang *et al.*, 2006; Ren *et al.*, 2008; Li *et al.*, 2006; Knoth *et al.*, 2007; von Saint Paul *et al.*, 2011
M1C12	AT2G38470	Transcription factor, JA pathway	Wan *et al.*, 2004; Libault *et al.*, 2007; Lippok *et al.*, 2007; Sarowar *et al.*, 2011; Birkenbihl *et al.*, 2012
M12E4	AT1G27500	Tetratricopeptide repeat	Tör *et al.*, 2002; Kwon *et al.*, 2009
M22A6	AT1G80360	Pyridoxal-phosphate, oxidative stress response	Denslow *et al.*, 2005
M14G2	AT4G39820	Tetratricopeptide repeat	Tör *et al.*, 2002; Kwon *et al.*, 2009
M8H2	AT5G64250	JA pathway	Armengaud *et al.*, 2004; Torres-Zabala *et al.*, 2007
M26D3	AT4G01100	Purine transporter, Signalling	Demidchik and Maathuis, 2007; Palmieri *et al.*, 2008
M25B1	AT3G13790	Cell wall invertase, signalling	Sherson *et al.*, 2003; Fotopoulos *et al.*, 2003; Verhaest *et al.*, 2005
M9E10	AT1G44750	Purine transporter, Signalling	Gillissen *et al.*, 2000; Demidchik and Maathuis, 2007
M23C11	AT1G76180	ABA responsive	Nylander *et al.*, 2001; BRAY, 2002; Kovacs *et al.*, 2008; Kline *et al.*, 2010
M16H1	no hit found	Auxin responsive	
M9D5	AT4G37150	SA release from MeSA	Manosalva *et al.*, 2010; Vlot *et al.*, 2008; Yang *et al.*, 2008
M30F8	AT1G28480	SA pathway, REDOX signaling	Rouhier *et al.*, 2006; Krinke *et al.*, 2007; Ghanta and author, 2011; Noctor *et al.*, 2011
M28A2	AT5G42650	JA synthesis	Peña-Cortés *et al.*, 2004; Leon-Reyes *et al.*, 2010

Table S3b. Down-regulated genes with predicted role in defence response against *C. acutatum* and associated references. A selection of references linking these genes to defence response in plants is showed. Color code of each group of genes is associated with functional classification as in Table I.

Strawberry gene ID	*A. thaliana* Orthologue	Relation with Defense/Function	References
**Invasion sensing**			
M6F8	AT1G57680	G-protein coupled receptor	Gilman, 1987; Gookin *et al.*, 2008
M20C3	AT2G32240	LRR protein, plant receptor	Kline *et al.*, 2010; Kaplan *et al.*, 2006
**Signal Transduction**			
M18F3	AT5G43010	Regulatory ATPase, Proteasome complex	
M29G3	AT2G22990	Peptidase, Glucosinolate and phenylpropanoid pathway	Beers *et al.*, 2004; Kliebenstein *et al.*, 2005; Kaschani *et al.*, 2009
M5E3	AT1G74960	Fatty acid biosynthesis	
M26F4	AT5G67090	Peptidase	Beers *et al.*, 2004
M22F5	AT5G02310	Ubiquitin ligase, Proteasome complex	Garzón *et al.*, 2007
**New Protein Synthesis and Secretion**			
M10H10	AT2G32700	Transcription represor	Consortium, 2011; Causier *et al.*, 2012; Shyu *et al.*, 2012
M28F7	AT5G02960	Protein synthesis	Baima *et al.*, 1995; Barakat *et al.*, 2001; Carroll *et al.*, 2008
M22E3	AT5G53430	Indirect transcription regulation	Springer *et al.*, 2003
M22E11	AT1G15750	Transcription represor	Consortium, 2011; Causier *et al.*, 2012; Shyu *et al.*, 2012
M22D5	AT1G22910	RNA metabolism	Peal *et al.*, 2011; De Paepe *et al.*, 2004; Pavet *et al.*, 2005; Lorković *et al.*, 2000
M21G2	AT1G29170	Citoesqueleton	Zhang *et al.*, 2008
**Direct Defences**			
M29H6	AT4G22880	Secondary metabolism	Martens *et al.*, 2010; Abrahams *et al.*, 2003; Shan *et al.*, 2009; Devoto *et al.*, 2005
M29C12	AT5G05270	Secondary metabolism	Wei *et al.*, 2006
M19C6	AT5G15870	Cell wall degradation, PR protein family	
M21F3	AT1G36370	REDOX production	Moreno *et al.*, 2005
**Hormone-Dependent Pathways**			

M18H1	AT1G07590	Tetratricopeptide repeat	Tör *et al.*, 2002; Kwon *et al.*, 2009
M15G5	AT4G03550	Negative regulator SA dependent defences	Adie *et al.*, 2007; Wawrzynska *et al.*, 2010

References

Abrahams S, Lee E, Walker AR, Tanner GJ, Larkin PJ, Ashton AR. 2003. The Arabidopsis TDS4 gene encodes leucoanthocyanidin dioxygenase (LDOX) and is essential for proanthocyanidin synthesis and vacuole development. *The Plant Journal* **35**, 624-636.

Abrash EB, Bergmann DC. 2010. Regional specification of stomatal production by the putative ligand CHALLAH. *Development* **137**, 447-455.

AbuQamar S, Chen X, Dhawan R, Bluhm B, Salmeron J, Lam S, Dietrich RA, Mengiste T. 2006. Expression profiling and mutant analysis reveals complex regulatory networks involved in Arabidopsis response to Botrytis infection. *The Plant Journal* **48**, 28-44.

Adie BAT, Perez-Perez J, Perez-Perez MM, Godoy M, Sanchez-Serrano J-J, Schmelz EA, Solano R. 2007. ABA Is an Essential Signal for Plant Resistance to Pathogens Affecting JA Biosynthesis and the Activation of Defenses in Arabidopsis. *Plant Cell*, tpc.106.048041.

Alvarez-Buylla ER, Benítez M, Corvera-Poiré A, Chaos Cador Á, de Folter S, Gamboa de Buen A, Garay-Arroyo A, García-Ponce B, Jaimes-Miranda F, Pérez-Ruiz RV, Piñeyro-Nelson A, Sánchez-Corrales YE. 2010. Flower Development. *The Arabidopsis Book*, e0127.

Amil-Ruiz F, Blanco-Portales R, Muñoz-Blanco J, Caballero JL. 2011. The Strawberry Plant Defence Mechanism: A Molecular Review. *Plant and Cell Physiology* **52**, 1873-1903.

Andersson-Gunnerås S, Mellerowicz EJ, Love J, Segerman B, Ohmiya Y, Coutinho PM, Nilsson P, Henrissat B, Moritz T, Sundberg B. 2006. Biosynthesis of cellulose-enriched tension wood in Populus: global analysis of transcripts and metabolites identifies biochemical and developmental regulators in secondary wall biosynthesis. *The Plant Journal* **45**, 144-165.

Armengaud P, Breitling R, Amtmann A. 2004. The Potassium-Dependent Transcriptome of Arabidopsis Reveals a Prominent Role of Jasmonic Acid in Nutrient Signaling. *Plant Physiology* **136**, 2556-2576.

Ascencio-Ibáñez JT, Sozzani R, Lee T-J, Chu T-M, Wolfinger RD, Cella R, Hanley-Bowdoin L. 2008. Global Analysis of Arabidopsis Gene Expression Uncovers a Complex Array of Changes Impacting Pathogen Response and Cell Cycle during Geminivirus Infection. *Plant Physiology* **148**, 436-454.

Ashfield T, Ong LE, Nobuta K, Schneider CM, Innes RW. 2004. Convergent Evolution of Disease Resistance Gene Specificity in Two Flowering Plant Families. *The Plant Cell Online* **16**, 309-318.

Bäckström S, Elfving N, Nilsson R, Wingsle G, Björklund S. 2007. Purification of a Plant Mediator from Arabidopsis thaliana Identifies PFT1 as the Med25 Subunit. *Molecular Cell* **26**, 717-729.

Baima S, Sessa G, Ruberti I, Morelli G. 1995. A cDNA encoding Arabidopsis thaliana cytoplasmic ribosomal protein L18. *Gene* **153**, 171-174.

Barakat A, Szick-Miranda K, Chang I-F, Guyot R, Blanc G, Cooke R, Delseny M, Bailey-Serres J. 2001. The Organization of Cytoplasmic Ribosomal Protein Genes in the Arabidopsis Genome. *Plant Physiology* **127**, 398-415.
Barkla BJ, Hirschi KD, Pittman JK. 2008. Exchangers man the pumps: Functional interplay between proton pumps and proton-coupled Ca²⁺ exchangers. *Plant Signaling & Behavior* **3**, 354-356.
Bassham DC, Brandizzi F, Otegui MS, Sanderfoot AA. 2008. The Secretory System of Arabidopsis. *The Arabidopsis Book*, e0116.
Beers EP, Jones AM, Dickerman AW. 2004. The S8 serine, C1A cysteine and A1 aspartic protease families in Arabidopsis. *Phytochemistry* **65**, 43-58.
Besson-Bard A, Pugin A, Wendehenne D. 2008. New Insights into Nitric Oxide Signaling in Plants. *Annual Review of Plant Biology* **59**, 21-39.
Bhattacharjee S, Halane MK, Kim SH, Gassmann W. 2011. Pathogen Effectors Target Arabidopsis EDS1 and Alter Its Interactions with Immune Regulators. *Science* **334**, 1405-1408.
Birkenbihl RP, Diezel C, Somssich IE. 2012. Arabidopsis WRKY33 Is a Key Transcriptional Regulator of Hormonal and Metabolic Responses toward Botrytis cinerea Infection. *Plant Physiology* **159**, 266-285.
Bíró J, Farkas I, Domoki M, Ötvös K, Bottka S, Dombrádi V, Fehér A. 2012. The histone phosphatase inhibitory property of plant nucleosome assembly protein-related proteins (NRPs). *Plant Physiology and Biochemistry* **52**, 162-168.
Blanco-Portales R, Medina-Escobar N, López-Ráez JA, González-Reyes JA, Villalba JM, Moyano E, Caballero JL, Muñoz-Blanco J. 2002. Cloning, expression and immunolocalization pattern of a cinnamyl alcohol dehydrogenase gene from strawberry (Fragaria × ananassa cv. Chandler). *Journal of Experimental Botany* **53**, 1723-1734.
Block-Schmidt AS, Dukowic-Schulze S, Wanieck K, Reidt W, Puchta H. 2011. BRCC36A is epistatic to BRCA1 in DNA crosslink repair and homologous recombination in Arabidopsis thaliana. *Nucleic Acids Research* **39**, 146-154.
Boisson B, Giglione C, Meinnel T. 2003. Unexpected Protein Families Including Cell Defense Components Feature in the N-Myristoylome of a Higher Eukaryote. *Journal of Biological Chemistry* **278**, 43418-43429.
Borner GHH, Sherrier DJ, Stevens TJ, Arkin IT, Dupree P. 2002. Prediction of Glycosylphosphatidylinositol-Anchored Proteins in Arabidopsis. A Genomic Analysis. *Plant Physiology* **129**, 486-499.
Borner GHH, Lilley KS, Stevens TJ, Dupree P. 2003. Identification of Glycosylphosphatidylinositol-Anchored Proteins in Arabidopsis. A Proteomic and Genomic Analysis. *Plant Physiology* **132**, 568-577.
BRAY EA. 2002. Classification of Genes Differentially Expressed during Water-deficit Stress in Arabidopsis thaliana: an Analysis using Microarray and Differential Expression Data. *Annals of Botany* **89**, 803-811.
Brodersen P, Petersen M, Bjørn Nielsen H, Zhu S, Newman MA, Shokat KM, Rietz S, Parker J, Mundy J. 2006. Arabidopsis MAP kinase 4 regulates salicylic acid- and jasmonic acid/ethylene-dependent responses via EDS1 and PAD4. *The Plant Journal* **47**, 532-546.
Caldwell KS, Michelmore RW. 2009. Arabidopsis thaliana Genes Encoding Defense Signaling and Recognition Proteins Exhibit Contrasting Evolutionary Dynamics. *Genetics* **181**, 671-684.
Callis J, Vierstra RD. 2000. Protein degradation in signaling. *Current Opinion in Plant Biology* **3**, 381-386.
Cannon SB, Zhu H, Baumgarten AM, Spangler R, Cook GMDR. 2002. Diversity, Distribution, and Ancient Taxonomic Relationships Within the TIR and Non-TIR NBS-LRR Resistance Gene Subfamilies. *Journal of Molecular Evolution* **54**, 548-562.
Carroll AJ, Heazlewood JL, Ito J, Millar AH. 2008. Analysis of the Arabidopsis Cytosolic Ribosome Proteome Provides Detailed Insights into Its Components and Their Post-translational Modification. *Molecular & Cellular Proteomics* **7**, 347-369.
Casado-Díaz A, Encinas-Villarejo S, Santos Bdl, Schilirò E, Yubero-Serrano E-M, Amil-Ruiz F, Pocovi MI, Pliego-Alfaro F, Dorado G, Rey M, Romero F, Muñoz-Blanco J, Caballero J-L. 2006. Analysis of strawberry genes differentially expressed in response to *Colletotrichum* infection. *Physiologia Plantarum* **128**, 633-650.

Causier B, Lloyd J, Stevens L, Davies B. 2012. TOPLESS co-repressor interactions and their evolutionary conservation in plants. *Plant Signaling & Behavior* **7**, 325-328.

Consortium AIM. 2011. Evidence for Network Evolution in an Arabidopsis Interactome Map. *Science* **333**, 601-607.

Chassin Y, Kapri-Pardes E, Sinvany G, Arad T, Adam Z. 2002. Expression and Characterization of the Thylakoid Lumen Protease DegP1 from Arabidopsis. *Plant Physiology* **130**, 857-864.

Cheng N-H, Pittman JK, Zhu J-K, Hirschi KD. 2004. The Protein Kinase SOS2 Activates the Arabidopsis H+/Ca2+ Antiporter CAX1 to Integrate Calcium Transport and Salt Tolerance. *Journal of Biological Chemistry* **279**, 2922-2926.

Cheng N-H, Pittman JK, Shigaki T, Lachmansingh J, LeClere S, Lahner B, Salt DE, Hirschi KD. 2005. Functional Association of Arabidopsis CAX1 and CAX3 Is Required for Normal Growth and Ion Homeostasis. *Plant Physiology* **138**, 2048-2060.

Chew O, Whelan J, Millar AH. 2003. Molecular Definition of the Ascorbate-Glutathione Cycle in Arabidopsis Mitochondria Reveals Dual Targeting of Antioxidant Defenses in Plants. *Journal of Biological Chemistry* **278**, 46869-46877.

Chiang C-S, Stacey G, Tsay Y-F. 2004. Mechanisms and Functional Properties of Two Peptide Transporters, AtPTR2 and fPTR2. *Journal of Biological Chemistry* **279**, 30150-30157.

Chivasa S, Hamilton JM, Pringle RS, Ndimba BK, Simon WJ, Lindsey K, Slabas AR. 2006. Proteomic analysis of differentially expressed proteins in fungal elicitor-treated Arabidopsis cell cultures. *Journal of Experimental Botany* **57**, 1553-1562.

Day B, He SY. 2010. Battling Immune Kinases in Plants. *Cell host & microbe* **7**, 259-261.

De Paepe A, Vuylsteke M, Van Hummelen P, Zabeau M, Van Der Straeten D. 2004. Transcriptional profiling by cDNA-AFLP and microarray analysis reveals novel insights into the early response to ethylene in Arabidopsis. *The Plant Journal* **39**, 537-559.

del Pozo JC, Dharmasiri S, Hellmann H, Walker L, Gray WM, Estelle M. 2002. AXR1-ECR1–Dependent Conjugation of RUB1 to the Arabidopsis Cullin AtCUL1 Is Required for Auxin Response. *The Plant Cell Online* **14**, 421-433.

Delauré SL, Van Hemelrijck W, De Bolle MFC, Cammue BPA, De Coninck BMA. 2008. Building up plant defenses by breaking down proteins. *Plant Science* **174**, 375-385.

Demidchik V, Maathuis FJM. 2007. Physiological roles of nonselective cation channels in plants: from salt stress to signalling and development. *New Phytologist* **175**, 387-404.

Denslow SA, Walls AA, Daub ME. 2005. Regulation of biosynthetic genes and antioxidant properties of vitamin B□ vitamers during plant defense responses. *Physiological and Molecular Plant Pathology* **66**, 244-255.

Devoto A, Ellis C, Magusin A, Chang H-S, Chilcott C, Zhu T, Turner J. 2005. Expression profiling reveals COI1 to be a key regulator of genes involved in wound- and methyl jasmonate-induced secondary metabolism, defence, and hormone interactions. *Plant Molecular Biology* **58**, 497-513.

DeYoung BJ, Qi D, Kim S-H, Burke TP, Innes RW. 2012. Activation of a plant nucleotide binding-leucine rich repeat disease resistance protein by a modified self protein. *Cellular Microbiology*, no-no.

Dharmasiri N, Dharmasiri S, Weijers D, Karunarathna N, Jurgens G, Estelle M. 2007. AXL and AXR1 have redundant functions in RUB conjugation and growth and development in Arabidopsis. *The Plant Journal* **52**, 114-123.

Dong X. 2004. NPR1, all things considered. *Current Opinion in Plant Biology* **7**, 547-552.

Du L, Chen Z. 2000. Identification of genes encoding receptor-like protein kinases as possible targets of pathogen- and salicylic acid-induced WRKY DNA-binding proteins in Arabidopsis. *The Plant Journal* **24**, 837-847.

Duchêne A-M, Giritch A, Hoffmann B, Cognat V, Lancelin D, Peeters NM, Zaepfel M, Maréchal-Drouard L, Small ID. 2005. Dual targeting is the rule for organellar aminoacyl-tRNA synthetases in Arabidopsis thaliana. *Proceedings of the National Academy of Sciences of the United States of America* **102**, 16484-16489.

Dunkley TPJ, Watson R, Griffin JL, Dupree P, Lilley KS. 2004. Localization of Organelle Proteins by Isotope Tagging (LOPIT). *Molecular & Cellular Proteomics* **3**, 1128-1134.
Encinas-Villarejo S, Maldonado AM, Amil-Ruiz F, de los Santos B, Romero F, Pliego-Alfaro F, Munoz-Blanco J, Caballero JL. 2009. Evidence for a positive regulatory role of strawberry (*Fragaria x ananassa*) Fa WRKY1 and Arabidopsis At WRKY75 proteins in resistance. *Journal of Experimental Botany* **60**, 3043-3065.
Englbrecht C, Schoof H, Bohm S. 2004. Conservation, diversification and expansion of C2H2 zinc finger proteins in the Arabidopsis thaliana genome. *BMC Genomics* **5**, 39.
Fabro G, Di Rienzo JA, Voigt CA, Savchenko T, Dehesh K, Somerville S, Alvarez ME. 2008. Genome-Wide Expression Profiling Arabidopsis at the Stage of Golovinomyces cichoracearum Haustorium Formation. *Plant Physiology* **146**, 1421-1439.
Ferrando A, Koncz-Kálmán Z, Farràs R, Tiburcio A, Schell J, Koncz C. 2001. Detection of in vivo protein interactions between Snf1-related kinase subunits with intron-tagged epitope-labelling in plants cells. *Nucleic Acids Research* **29**, 3685-3693.
Fotopoulos V, Gilbert MJ, Pittman JK, Marvier AC, Buchanan AJ, Sauer N, Hall JL, Williams LE. 2003. The Monosaccharide Transporter Gene, AtSTP4, and the Cell-Wall Invertase, Atβfruct1, Are Induced in Arabidopsis during Infection with the Fungal Biotroph Erysiphe cichoracearum. *Plant Physiology* **132**, 821-829.
Garzón M, Eifler K, Faust A, Scheel H, Hofmann K, Koncz C, Yephremov A, Bachmair A. 2007. PRT6/At5g02310 encodes an Arabidopsis ubiquitin ligase of the N-end rule pathway with arginine specificity and is not the CER3 locus. *FEBS Letters* **581**, 3189-3196.
Ghanta S, author SC. 2011. Glutathione as a signaling molecule. Another challenge to pathogens. *Plant Signaling and Behavior* **6**, 783–788.
Gilman AG. 1987. G Proteins: Transducers of Receptor-Generated Signals. *Annual Review of Biochemistry* **56**, 615-649.
Gillissen B, Bürkle L, André B, Kühn C, Rentsch D, Brandl B, Frommer WB. 2000. A New Family of High-Affinity Transporters for Adenine, Cytosine, and Purine Derivatives in Arabidopsis. *The Plant Cell Online* **12**, 291-300.
Gookin T, Kim J, Assmann S. 2008. Whole proteome identification of plant candidate G-protein coupled receptors in Arabidopsis, rice, and poplar: computational prediction and in-vivo protein coupling. *Genome Biology* **9**, R120.
Gou M, Su N, Zheng J, Huai J, Wu G, Zhao J, He J, Tang D, Yang S, Wang G. 2009. An F-box gene, CPR30, functions as a negative regulator of the defense response in Arabidopsis. *The Plant Journal* **60**, 757-770.
Gracey AY, Troll JV, Somero GN. 2001. Hypoxia-induced gene expression profiling in the euryoxic fish Gillichthys mirabilis. *Proceedings of the National Academy of Sciences* **98**, 1993-1998.
Heidrich K, Wirthmueller L, Tasset C, Pouzet C, Deslandes L, Parker JE. 2011. Arabidopsis EDS1 Connects Pathogen Effector Recognition to Cell Compartment–Specific Immune Responses. *Science* **334**, 1401-1404.
Himmelbach A, Hoffmann T, Leube M, Hohener B, Grill E. 2002. Homeodomain protein ATHB6 is a target of the protein phosphatase ABI1 and regulates hormone responses in Arabidopsis. *EMBO J* **21**, 3029-3038.
Hirano T, Matsuzawa T, Takegawa K, Sato MH. 2011. Loss-of-Function and Gain-of-Function Mutations in FAB1A/B Impair Endomembrane Homeostasis, Conferring Pleiotropic Developmental Abnormalities in Arabidopsis. *Plant Physiology* **155**, 797-807.
Hirano T, Sato MH. 2011. Arabidopsis *FAB1A/B* is possibly involved in the recycling of auxin transporters. *Plant Signaling & Behavior* **6**, 583-585.
Holst K, Schmülling T, Werner T. 2011. Enhanced cytokinin degradation in leaf primordia of transgenic Arabidopsis plants reduces leaf size and shoot organ primordia formation. *Journal of Plant Physiology* **168**, 1328-1334.
Hotton S, Eigenheer R, Castro M, Bostick M, Callis J. 2011. AXR1-ECR1 and AXL1-ECR1 heterodimeric RUB-activating enzymes diverge in function in *Arabidopsis thaliana*. *Plant Molecular Biology* **75**, 515-526.

Hrabak EM, Chan CWM, Gribskov M, Harper JF, Choi JH, Halford N, Kudla J, Luan S, Nimmo HG, Sussman MR, Thomas M, Walker-Simmons K, Zhu J-K, Harmon AC. 2003. The Arabidopsis CDPK-SnRK Superfamily of Protein Kinases. *Plant Physiology* **132**, 666-680.
Hu Y, Dong Q, Yu D. 2012. Arabidopsis WRKY46 coordinates with WRKY70 and WRKY53 in basal resistance against pathogen Pseudomonas syringae. *Plant Science* **185–186**, 288-297.
Huang S, An Y-Q, McDowell JM, McKinney EC, Meagher RB. 1997. The Arabidopsis ACT11 actin gene is strongly expressed in tissues of the emerging inflorescence, pollen, and developing ovules. *Plant Molecular Biology* **33**, 125-139.
Joe Louis K-LK, Vijay Singh, John Reese, Jyoti Shah. 2011. Arabidopsis MPL (MYZUS PERSICAE INDUCED LIPASE) Mediated Resistance Against Green Peach Aphid. *22ND INTERNATIONAL CONFERENCE ON ARABIDOPSIS RESEARCH*.
Kamauchi S, Nakatani H, Nakano C, Urade R. 2005. Gene expression in response to endoplasmic reticulum stress in Arabidopsis thaliana. *FEBS Journal* **272**, 3461-3476.
Kaplan B, Davydov O, Knight H, Galon Y, Knight MR, Fluhr R, Fromm H. 2006. Rapid Transcriptome Changes Induced by Cytosolic Ca2+ Transients Reveal ABRE-Related Sequences as Ca2+-Responsive cis Elements in Arabidopsis. *The Plant Cell Online* **18**, 2733-2748.
Kaschani F, Gu C, Niessen S, Hoover H, Cravatt BF, van der Hoorn RAL. 2009. Diversity of Serine Hydrolase Activities of Unchallenged and Botrytis-infected Arabidopsis thaliana. *Molecular & Cellular Proteomics* **8**, 1082-1093.
Katagiri T, Takahashi S, Shinozaki K. 2001. Involvement of a novel Arabidopsis phospholipase D, AtPLDδ, in dehydration-inducible accumulation of phosphatidic acid in stress signalling. *The Plant Journal* **26**, 595-605.
Khan AA, Shi Y, Shih DS. 2003. Cloning and partial characterization of a β-1,3-glucanase gene from strawberry. *Mitochondrial DNA* **14**, 406-412.
Kiba T, Naitou T, Koizumi N, Yamashino T, Sakakibara H, Mizuno T. 2005. Combinatorial Microarray Analysis Revealing Arabidopsis Genes Implicated in Cytokinin Responses through the His→Asp Phosphorelay Circuitry. *Plant and Cell Physiology* **46**, 339-355.
Kim MJ, Ciani S, Schachtman DP. 2010. A Peroxidase Contributes to ROS Production during Arabidopsis Root Response to Potassium Deficiency. *Molecular Plant* **3**, 420-427.
Kim TKHaJ-S. 2011. Genomic identification of putative allergen genes in woodland strawberry (Fragaria vesca) and mandarin orange (Citrus clementina). *Plant Omics Journal* **4**, 428-434.
Kliebenstein DJ, Rowe HC, Denby KJ. 2005. Secondary metabolites influence Arabidopsis/Botrytis interactions: variation in host production and pathogen sensitivity. *The Plant Journal* **44**, 25-36.
Kline KG, Barrett-Wilt GA, Sussman MR. 2010. In planta changes in protein phosphorylation induced by the plant hormone abscisic acid. *Proceedings of the National Academy of Sciences* **107**, 15986-15991.
Knoth C, Ringler J, Dangl JL, Eulgem T. 2007. Arabidopsis WRKY70 Is Required for Full RPP4-Mediated Disease Resistance and Basal Defense Against Hyaloperonospora parasitica. *Molecular Plant-Microbe Interactions* **20**, 120-128.
Ko J-H, Yang SH, Park AH, Lerouxel O, Han K-H. 2007. ANAC012, a member of the plant-specific NAC transcription factor family, negatively regulates xylary fiber development in Arabidopsis thaliana. *The Plant Journal* **50**, 1035-1048.
Kolukisaoglu Ü, Weinl S, Blazevic D, Batistic O, Kudla J. 2004. Calcium Sensors and Their Interacting Protein Kinases: Genomics of the Arabidopsis and Rice CBL-CIPK Signaling Networks. *Plant Physiology* **134**, 43-58.
Kosarev P, Mayer K, Hardtke C. 2002. Evaluation and classification of RING-finger domains encoded by the Arabidopsis genome. *Genome Biology* **3**, research0016.0011 - research0016.0012.
Kovacs D, Kalmar E, Torok Z, Tompa P. 2008. Chaperone Activity of ERD10 and ERD14, Two Disordered Stress-Related Plant Proteins. *Plant Physiology* **147**, 381-390.

Kraft E, Stone SL, Ma L, Su N, Gao Y, Lau O-S, Deng X-W, Callis J. 2005. Genome Analysis and Functional Characterization of the E2 and RING-Type E3 Ligase Ubiquitination Enzymes of Arabidopsis. *Plant Physiology* **139**, 1597-1611.
Kranz HD, Denekamp M, Greco R, Jin H, Leyva A, Meissner RC, Petroni K, Urzainqui A, Bevan M, Martin C, Smeekens S, Tonelli C, Paz-Ares J, Weisshaar B. 1998. Towards functional characterisation of the members of theR2R3-MYBgene family fromArabidopsis thaliana. *The Plant Journal* **16**, 263-276.
Krinke O, Ruelland E, Valentová O, Vergnolle C, Renou J-P, Taconnat L, Flemr M, Burketová L, Zachowski A. 2007. Phosphatidylinositol 4-Kinase Activation Is an Early Response to Salicylic Acid in Arabidopsis Suspension Cells. *Plant Physiology* **144**, 1347-1359.
Krizek B. 2011. Aintegumenta and Aintegumenta-Like6 regulate auxin-mediated flower development in Arabidopsis. *BMC Research Notes* **4**, 176.
Krizek B, Eaddy M. 2012. *AINTEGUMENTA LIKE6* regulates cellular differentiation in flowers. *Plant Molecular Biology* **78**, 199-209.
Krizek BA, Prost V, Macias A. 2000. AINTEGUMENTA Promotes Petal Identity and Acts as a Negative Regulator of AGAMOUS. *The Plant Cell Online* **12**, 1357-1366.
Kwon C, Bednarek P, Schulze-Lefert P. 2008. Secretory pathways in plant immune responses. *Plant Physiology* **147**, 1575-1583.
Kwon SI, Kim SH, Bhattacharjee S, Noh J-J, Gassmann W. 2009. SRFR1, a suppressor of effector-triggered immunity, encodes a conserved tetratricopeptide repeat protein with similarity to transcriptional repressors. *The Plant Journal* **57**, 109-119.
Langlois-Meurinne M, Gachon CMM, Saindrenan P. 2005. Pathogen-Responsive Expression of Glycosyltransferase Genes UGT73B3 and UGT73B5 Is Necessary for Resistance to Pseudomonas syringae pv tomato in Arabidopsis. *Plant Physiology* **139**, 1890-1901.
Lasserre E, Jobet E, Llauro C, Delseny M. 2008. AtERF38 (At2g35700), an AP2/ERF family transcription factor gene from Arabidopsis thaliana, is expressed in specific cell types of roots, stems and seeds that undergo suberization. *Plant Physiology and Biochemistry* **46**, 1051-1061.
Lee AH-Y, Hurley B, Felsensteiner C, Yea C, Ckurshumova W, Bartetzko V, Wang PW, Quach V, Lewis JD, Liu YC, Börnke F, Angers S, Wilde A, Guttman DS, Desveaux D. 2012. A Bacterial Acetyltransferase Destroys Plant Microtubule Networks and Blocks Secretion. *PLoS Pathog* **8**, e1002523.
Lee Y-RJ, Liu B. 2004. Cytoskeletal Motors in Arabidopsis. Sixty-One Kinesins and Seventeen Myosins. *Plant Physiology* **136**, 3877-3883.
Lee Y, Bak G, Choi Y, Chuang W-I, Cho H-T, Lee Y. 2008. Roles of Phosphatidylinositol 3-Kinase in Root Hair Growth. *Plant Physiology* **147**, 624-635.
Leon-Reyes A, Van der Does D, De Lange E, Delker C, Wasternack C, Van Wees S, Ritsema T, Pieterse C. 2010. Salicylate-mediated suppression of jasmonate-responsive gene expression in Arabidopsis is targeted downstream of the jasmonate biosynthesis pathway. *Planta* **232**, 1423-1432.
Leshem Y, Seri L, Levine A. 2007. Induction of phosphatidylinositol 3-kinase-mediated endocytosis by salt stress leads to intracellular production of reactive oxygen species and salt tolerance. *The Plant Journal* **51**, 185-197.
Li D, Liu H, Zhang H, Wang X, Song F. 2008. OsBIRH1, a DEAD-box RNA helicase with functions in modulating defence responses against pathogen infection and oxidative stress. *Journal of Experimental Botany* **59**, 2133-2146.
Li J, Brader G, Palva ET. 2004. The WRKY70 transcription factor: A node of convergence for Jasmonate-mediated and Salicylate-mediated signals in plant defense. *Plant Cell* **16**, 319-331.
Li J, Brader G, Kariola T, Tapio Palva E. 2006. WRKY70 modulates the selection of signaling pathways in plant defense. *The Plant Journal* **46**, 477-491.
Libault M, Wan J, Czechowski T, Udvardi M, Stacey G. 2007. Identification of 118 Arabidopsis Transcription Factor and 30 Ubiquitin-Ligase Genes Responding to Chitin, a Plant-Defense Elicitor. *Molecular Plant-Microbe Interactions* **20**, 900-911.
Linder P, Owttrim GW. 2009. Plant RNA helicases: linking aberrant and silencing RNA. *Trends in Plant Science* **14**, 344-352.
Lippok B, Birkenbihl RP, Rivory G, Brümmer J, Schmelzer E, Logemann E, Somssich IE. 2007. Expression of AtWRKY33 encoding a Pathogen- or PAMP-Responsive WRKY transcription factor is regulated by a composite DNA motif containing W box elements. *Molecular Plant-Microbe Interactions* **20**, 420-429.
Lohmann D, Stacey N, Breuninger H, Jikumaru Y, Müller D, Sicard A, Leyser O, Yamaguchi S, Lenhard M. 2010. SLOW MOTION Is Required for Within-Plant Auxin Homeostasis and Normal Timing of Lateral Organ Initiation at the Shoot Meristem in Arabidopsis. *The Plant Cell Online* **22**, 335-348.

Lorenzo O, Solano R. 2005. Molecular players regulating the jasmonate signalling network. *Current Opinion in Plant Biology* **8**, 532-540.
Lorković ZJ, Wieczorek Kirk DA, Klahre U, Hemmings-Mieszczak M, Filipowicz W. 2000. RBP45 and RBP47, two oligouridylate-specific hnRNP-like proteins interacting with poly(A)+ RNA in nuclei of plant cells. *RNA* **6**, 1610-1624.
Losa A, Colombo M, Brambilla V, Colombo L. 2010. Genetic interaction between AINTEGUMENTA (ANT) and the ovule identity genes SEEDSTICK (STK), SHATTERPROOF1 (SHP1) and SHATTERPROOF2 (SHP2). *Sexual Plant Reproduction* **23**, 115-121.
Louis J, Leung Q, Pegadaraju V, Reese J, Shah J. 2010a. PAD4-Dependent Antibiosis Contributes to the ssi2-Conferred Hyper-Resistance to the Green Peach Aphid. *Molecular Plant-Microbe Interactions* **23**, 618-627.
Louis J, Lorenc-Kukula K, Singh V, Reese J, Jander G, Shah J. 2010b. Antibiosis against the green peach aphid requires the Arabidopsis thaliana MYZUS PERSICAE-INDUCED LIPASE1 gene. *The Plant Journal* **64**, 800-811.
Louis J, Gobbato E, Mondal HA, Feys BJ, Parker JE, Shah J. 2012. Discrimination of Arabidopsis PAD4 Activities in Defense against Green Peach Aphid and Pathogens. *Plant Physiology* **158**, 1860-1872.
Lu L, Lee Y-RJ, Pan R, Maloof JN, Liu B. 2005. An Internal Motor Kinesin Is Associated with the Golgi Apparatus and Plays a Role in Trichome Morphogenesis in Arabidopsis. *Molecular Biology of the Cell* **16**, 811-823.
Mano Ji, Belles-Boix E, Babiychuk E, Inzé D, Torii Y, Hiraoka E, Takimoto K, Slooten L, Asada K, Kushnir S. 2005. Protection against Photooxidative Injury of Tobacco Leaves by 2-Alkenal Reductase. Detoxication of Lipid Peroxide-Derived Reactive Carbonyls. *Plant Physiology* **139**, 1773-1783.
Manohar M, Shigaki T, Mei H, Park S, Marshall J, Aguilar J, Hirschi KD. 2011. Characterization of Arabidopsis Ca2+/H+ Exchanger CAX3. *Biochemistry* **50**, 6189-6195.
Manosalva PM, Park S-W, Forouhar F, Tong L, Fry WE, Klessig DF. 2010. Methyl Esterase 1 (StMES1) Is Required for Systemic Acquired Resistance in Potato. *Molecular Plant-Microbe Interactions* **23**, 1151-1163.
Martens S, Preuß A, Matern U. 2010. Multifunctional flavonoid dioxygenases: Flavonol and anthocyanin biosynthesis in Arabidopsis thaliana L. *Phytochemistry* **71**, 1040-1049.
McGee JD, Roe JL, Sweat TA, Wang X, Guikema JA, Leach JE. 2003. Rice Phospholipase D Isoforms Show Differential Cellular Location and Gene Induction. *Plant and Cell Physiology* **44**, 1013-1026.
McKinney EC, Kandasamy MK, Meagher RB. 2002. Arabidopsis Contains Ancient Classes of Differentially Expressed Actin-Related Protein Genes. *Plant Physiology* **128**, 997-1007.
Merlet J, Burger J, Gomes JE, Pintard L. 2009. Regulation of cullin-RING E3 ubiquitin-ligases by neddylation and dimerization. *Cellular and Molecular Life Sciences* **66**, 1924-1938.
Meyers BC, Kozik A, Griego A, Kuang H, Michelmore RW. 2003. Genome-Wide Analysis of NBS-LRR-Encoding Genes in Arabidopsis. *The Plant Cell Online* **15**, 809-834.
Mladek C, Guger K, Hauser M-T. 2003. Identification and Characterization of the ARIADNEGene Family in Arabidopsis. A Group of Putative E3 Ligases. *Plant Physiology* **131**, 27-40.
Moreno JI, Martín R, Castresana C. 2005. Arabidopsis SHMT1, a serine hydroxymethyltransferase that functions in the photorespiratory pathway influences resistance to biotic and abiotic stress. *The Plant Journal* **41**, 451-463.
Moriyama E, Strope P, Opiyo S, Chen Z, Jones A. 2006. Mining the Arabidopsis thaliana genome for highly-divergent seven transmembrane receptors. *Genome Biology* **7**, R96.
Noctor G, Queval G, Mhamdi A, Chaouch S, Foyer CH. 2011. Glutathione. *The Arabidopsis Book*, e0142.

Nylander M, Svensson J, Palva ET, Welin BV. 2001. Stress-induced accumulation and tissue-specific localization of dehydrins in Arabidopsis thaliana. *Plant Molecular Biology* **45**, 263-279.
Ok SH, Jeong HJ, Bae JM, Shin J-S, Luan S, Kim K-N. 2005. Novel CIPK1-Associated Proteins in Arabidopsis Contain an Evolutionarily Conserved C-Terminal Region That Mediates Nuclear Localization. *Plant Physiology* **139**, 138-150.
Osorio S, Castillejo C, Quesada MA, Medina-Escobar N, Brownsey GJ, Suau R, Heredia A, Botella MA, Valpuesta V. 2008. Partial demethylation of oligogalacturonides by pectin methyl esterase 1 is required for eliciting defence responses in wild strawberry (*Fragaria vesca*). *The Plant Journal* **54**, 43-55.
Pajerowska-Mukhtar Karolina M, Wang W, Tada Y, Oka N, Tucker Chandra L, Fonseca Jose P, Dong X. 2012. The HSF-like Transcription Factor TBF1 Is a Major Molecular Switch for Plant Growth-to-Defense Transition. *Current biology : CB* **22**, 103-112.
Palmieri L, Santoro A, Carrari F, Blanco E, Nunes-Nesi A, Arrigoni R, Genchi F, Fernie AR, Palmieri F. 2008. Identification and Characterization of ADNT1, a Novel Mitochondrial Adenine Nucleotide Transporter from Arabidopsis. *Plant Physiology* **148**, 1797-1808.
Parker JE, Holub EB, Frost LN, Falk A, Gunn ND, Daniels MJ. 1996. Characterization of eds1, a mutation in Arabidopsis suppressing resistance to Peronospora parasitica specified by several different RPP genes. *The Plant Cell Online* **8**, 2033-2046.
Pavet V, Olmos E, Kiddle G, Mowla S, Kumar S, Antoniw J, Alvarez ME, Foyer CH. 2005. Ascorbic Acid Deficiency Activates Cell Death and Disease Resistance Responses in Arabidopsis. *Plant Physiology* **139**, 1291-1303.
Peal L, Jambunathan N, Mahalingam R. 2011. Phylogenetic and expression analysis of RNA-binding proteins with triple RNA recognition motifs in plants. *Molecules and Cells* **31**, 55-64.
Peer M, Stegmann M, Mueller MJ, Waller F. 2010. Pseudomonas syringae infection triggers de novo synthesis of phytosphingosine from sphinganine in Arabidopsis thaliana. *FEBS Letters* **584**, 4053-4056.
Peña-Cortés H, Barrios P, Dorta F, Polanco V, Sánchez C, Sánchez E, Ramírez I. 2004. Involvement of Jasmonic Acid and Derivatives in Plant Response to Pathogen and Insects and in Fruit Ripening. *Journal of Plant Growth Regulation* **23**, 246-260.
Pourcel L, Routaboul J-M, Kerhoas L, Caboche M, Lepiniec L, Debeaujon I. 2005. TRANSPARENT TESTA10 Encodes a Laccase-Like Enzyme Involved in Oxidative Polymerization of Flavonoids in Arabidopsis Seed Coat. *The Plant Cell Online* **17**, 2966-2980.
Qi Q, Rajala RVS, Anderson W, Jiang C, Rozwadowski K, Selvaraj G, Sharma R, Datla R. 2000. Molecular Cloning, Genomic Organization, and Biochemical Characterization of Myristoyl-CoA:ProteinN-Myristoyltransferase from Arabidopsis thaliana. *Journal of Biological Chemistry* **275**, 9673-9683.
Ren C-M, Zhu Q, Gao B-D, Ke S-Y, Yu W-C, Xie D-X, Peng W. 2008. Transcription Factor WRKY70 Displays Important but No Indispensable Roles in Jasmonate and Salicylic Acid Signaling. *Journal of Integrative Plant Biology* **50**, 630-637.
Rietz S, Stamm A, Malonek S, Wagner S, Becker D, Medina-Escobar N, Corina Vlot A, Feys BJ, Niefind K, Parker JE. 2011. Different roles of Enhanced Disease Susceptibility1 (EDS1) bound to and dissociated from Phytoalexin Deficient4 (PAD4) in Arabidopsis immunity. *New Phytologist* **191**, 107-119.
Rouhier N, Couturier J, Jacquot J-P. 2006. Genome-wide analysis of plant glutaredoxin systems. *Journal of Experimental Botany* **57**, 1685-1696.
Rustérucci C, Aviv DH, Holt BF, Dangl JL, Parker JE. 2001. The Disease Resistance Signaling Components EDS1 and PAD4 Are Essential Regulators of the Cell Death Pathway Controlled by LSD1 in Arabidopsis. *The Plant Cell Online* **13**, 2211-2224.
Ryu MY, Cho SK, Kim WT. 2010. The Arabidopsis C3H2C3-Type RING E3 Ubiquitin Ligase AtAIRP1 Is a Positive Regulator of an Abscisic Acid-Dependent Response to Drought Stress. *Plant Physiology* **154**, 1983-1997.
Samuel MA, Mudgil Y, Salt JN, Delmas F, Ramachandran S, Chilelli A, Goring DR. 2008. Interactions between the S-Domain Receptor Kinases and AtPUB-ARM E3 Ubiquitin Ligases Suggest a Conserved Signaling Pathway in Arabidopsis. *Plant Physiology* **147**, 2084-2095.

Sarowar S, Zhao Y, Soria-Guerra RE, Ali S, Zheng D, Wang D, Korban SS. 2011. Expression profiles of differentially regulated genes during the early stages of apple flower infection with Erwinia amylovora. *Journal of Experimental Botany* **62**, 4851-4861.
Schenk PM, Kazan K, Manners JM, Anderson JP, Simpson RS, Wilson IW, Somerville SC, Maclean DJ. 2003. Systemic Gene Expression in Arabidopsis during an Incompatible Interaction with Alternaria brassicicola. *Plant Physiology* **132**, 999-1010.
Schwartz BW, Yeung EC, Meinke DW. 1994. Disruption of morphogenesis and transformation of the suspensor in abnormal suspensor mutants of Arabidopsis. *Development* **120**, 3235-3245.
Shan X, Zhang Y, Peng W, Wang Z, Xie D. 2009. Molecular mechanism for jasmonate-induction of anthocyanin accumulation in Arabidopsis. *Journal of Experimental Botany* **60**, 3849-3860.
Shao F, Golstein C, Ade J, Stoutemyer M, Dixon JE, Innes RW. 2003. Cleavage of Arabidopsis PBS1 by a Bacterial Type III Effector. *Science* **301**, 1230-1233.
Sherson SM, Alford HL, Forbes SM, Wallace G, Smith SM. 2003. Roles of cell□wall invertases and monosaccharide transporters in the growth and development of Arabidopsis. *Journal of Experimental Botany* **54**, 525-531.
Shi Y, Zhang Y, Shih DS. 2006. Cloning and expression analysis of two [beta]-1,3-glucanase genes from strawberry. *Journal of Plant Physiology* **163**, 956-967.
Shimada C, Lipka V, O'Connell R, Okuno T, Schulze-Lefert P, Takano Y. 2006. Nonhost Resistance in Arabidopsis-Colletotrichum Interactions Acts at the Cell Periphery and Requires Actin Filament Function. *Molecular Plant-Microbe Interactions* **19**, 270-279.
Shimada T, Sugano S, Hara-Nishimura I. 2011. Positive and negative peptide signals control stomatal density. *Cellular and Molecular Life Sciences* **68**, 2081-2088.
Shyu C, Figueroa P, DePew CL, Cooke TF, Sheard LB, Moreno JE, Katsir L, Zheng N, Browse J, Howe GA. 2012. JAZ8 Lacks a Canonical Degron and Has an EAR Motif That Mediates Transcriptional Repression of Jasmonate Responses in Arabidopsis. *The Plant Cell Online* **24**, 536-550.
Simpson C, Thomas C, Findlay K, Bayer E, Maule AJ. 2009. An Arabidopsis GPI-Anchor Plasmodesmal Neck Protein with Callose Binding Activity and Potential to Regulate Cell-to-Cell Trafficking. *The Plant Cell Online* **21**, 581-594.
Sinvany-Villalobo G, Davydov O, Ben-Ari G, Zaltsman A, Raskind A, Adam Z. 2004. Expression in Multigene Families. Analysis of Chloroplast and Mitochondrial Proteases. *Plant Physiology* **135**, 1336-1345.
Smith ZR, Long JA. 2010. Control of Arabidopsis apical-basal embryo polarity by antagonistic transcription factors. *Nature* **464**, 423-426.
Snustad DP, Haas NA, Kopczak SD, Silflow CD. 1992. The small genome of Arabidopsis contains at least nine expressed beta-tubulin genes. *The Plant Cell Online* **4**, 549-556.
Söderman E, Mattsson J, Svenson M, Borkird C, Engström P. 1994. Expression patterns of novel genes encoding homeodomain leucine-zipper proteins in Arabidopsis thaliana. *Plant Molecular Biology* **26**, 145-154.
Son O, Cho H-Y, Kim M-R, Lee H, Lee M-S, Song E, Park JH, Nam KH, Chun J-Y, Kim H-J, Hong S-K, Chung Y-Y, Hur C-G, Cho H-T, Cheon C-I. 2004. Induction of a homeodomain–leucine zipper gene by auxin is inhibited by cytokinin in Arabidopsis roots. *Biochemical and Biophysical Research Communications* **326**, 203-209.
Springer NM, Napoli CA, Selinger DA, Pandey R, Cone KC, Chandler VL, Kaeppler HF, Kaeppler SM. 2003. Comparative Analysis of SET Domain Proteins in Maize and Arabidopsis Reveals Multiple Duplications Preceding the Divergence of Monocots and Dicots. *Plant Physiology* **132**, 907-925.
Stone SL, Hauksdóttir H, Troy A, Herschleb J, Kraft E, Callis J. 2005. Functional Analysis of the RING-Type Ubiquitin Ligase Family of Arabidopsis. *Plant Physiology* **137**, 13-30.
Swiderski MR, Innes RW. 2001. The Arabidopsis PBS1 resistance gene encodes a member of a novel protein kinase subfamily. *The Plant Journal* **26**, 101-112.
Tague BW, Goodman HM. 1995. Characterization of a family of Arabidopsis zinc finger protein cDNAs. *Plant Molecular Biology* **28**, 267-279.

Takemoto D, Rafiqi M, Hurley U, Lawrence GJ, Bernoux M, Hardham AR, Ellis JG, Dodds PN, Jones DA. 2011. N-Terminal Motifs in Some Plant Disease Resistance Proteins Function in Membrane Attachment and Contribute to Disease Resistance. *Molecular Plant-Microbe Interactions* **25**, 379-392.
Takenaka Y, Nakano S, Tamoi M, Sakuda S, Fukamizo T. 2009. Chitinase Gene Expression in Response to Environmental Stresses in *Arabidopsis thaliana*: Chitinase Inhibitor Allosamidin Enhances Stress Tolerance. *Bioscience, Biotechnology, and Biochemistry* **73**, 1066-1071.
Tamaoki M, Nakajima N, Kubo A, Aono M, Matsuyama T, Saji H. 2003. Transcriptome analysis of O3-exposed Arabidopsis reveals that multiple signal pathways act mutually antagonistically to induce gene expression. *Plant Molecular Biology* **53**, 443-456.
Tan X, Meyers B, Kozik A, West M, Morgante M, St Clair D, Bent A, Michelmore R. 2007. Global expression analysis of nucleotide binding site-leucine rich repeat-encoding and related genes in Arabidopsis. *BMC Plant Biology* **7**, 56.
Thilmony R, Underwood W, He SY. 2006. Genome-wide transcriptional analysis of the Arabidopsis thaliana interaction with the plant pathogen Pseudomonas syringae pv. tomato DC3000 and the human pathogen Escherichia coli O157:H7. *The Plant Journal* **46**, 34-53.
Thimm O, Bläsing O, Gibon Y, Nagel A, Meyer S, Krüger P, Selbig J, Müller LA, Rhee SY, Stitt M. 2004. MapMan: a user-driven tool to display genomics data sets onto diagrams of metabolic pathways and other biological processes. *The Plant Journal* **37**, 914-939.
Tischner R, Galli M, Heimer YM, Bielefeld S, Okamoto M, Mack A, Crawford NM. 2007. Interference with the citrulline-based nitric oxide synthase assay by argininosuccinate lyase activity in Arabidopsis extracts. *FEBS Journal* **274**, 4238-4245.
Tör M, Gordon P, Cuzick A, Eulgem T, Sinapidou E, Mert-Türk F, Can C, Dangl JL, Holub EB. 2002. Arabidopsis SGT1b Is Required for Defense Signaling Conferred by Several Downy Mildew Resistance Genes. *The Plant Cell Online* **14**, 993-1003.
Torres-Zabala Md, Truman W, Bennett MH, Lafforgue G, Mansfield JW, Rodriguez Egea P, Bogre L, Grant M. 2007. Pseudomonas syringae pv. tomato hijacks the Arabidopsis abscisic acid signalling pathway to cause disease. *EMBO J* **26**, 1434-1443.
Trotta A, Konert G, Rahikainen M, Aro E-M, Kangasjärvi S. 2011a. Knock-down of protein phosphatase 2A subunit B'γ promotes phosphorylation of CALRETICULIN 1 in Arabidopsis thaliana. *Plant Signal Behav* **6**, 1665–1668.
Trotta A, Wrzaczek M, Scharte J, Tikkanen M, Konert G, Rahikainen M, Holmström M, Hiltunen H-M, Rips S, Sipari N, Mulo P, Weis E, von Schaewen A, Aro E-M, Kangasjärvi S. 2011b. Regulatory Subunit B′γ of Protein Phosphatase 2A Prevents Unnecessary Defense Reactions under Low Light in Arabidopsis. *Plant Physiology* **156**, 1464-1480.
Uraji M, Katagiri T, Okuma E, Ye W, Hossain MA, Masuda C, Miura A, Nakamura Y, Mori IC, Shinozaki K, Murata Y. 2012. Cooperative Function of PLDδ and PLDα1 in Abscisic Acid-Induced Stomatal Closure in Arabidopsis. *Plant Physiology* **159**, 450-460.
Vassileva VN, Fujii Y, Ridge RW. 2005. Microtubule dynamics in plants. *Plant Biotechnology* **22**, 171-178.
Verhaest M, Le Roy K, Sansen S, De Coninck B, Lammens W, De Ranter CJ, Van Laere A, Van den Ende W, Rabijns A. 2005. Crystallization and preliminary X-ray diffraction study of a cell-wall invertase from Arabidopsis thaliana. *Acta Crystallographica Section F* **61**, 766-768.
Vlot AC, Liu P-P, Cameron RK, Park S-W, Yang Y, Kumar D, Zhou F, Padukkavidana T, Gustafsson C, Pichersky E, Klessig DF. 2008. Identification of likely orthologs of tobacco salicylic acid-binding protein 2 and their role in systemic acquired resistance in Arabidopsis thaliana. *The Plant Journal* **56**, 445-456.
von Saint Paul V, Zhang W, Kanawati B, Geist B, Faus-Keßler T, Schmitt-Kopplin P, Schäffner AR. 2011. The Arabidopsis Glucosyltransferase UGT76B1 Conjugates Isoleucic Acid and Modulates Plant Defense and Senescence. *The Plant Cell Online* **23**, 4124-4145.
Wan J, Zhang S, Stacey G. 2004. Activation of a mitogen-activated protein kinase pathway in Arabidopsis by chitin. *Molecular Plant Pathology* **5**, 125-135.
Wang D, Weaver ND, Kesarwani M, Dong X. 2005. Induction of Protein Secretory Pathway Is Required for Systemic Acquired Resistance. *Science* **308**, 1036-1040.
Wang D, Amornsiripanitch N, Dong X. 2006. A Genomic Approach to Identify Regulatory Nodes in the Transcriptional Network of Systemic Acquired Resistance in Plants. *PLoS Pathog* **2**, e123.

Wang D, Dong X. 2011. A Highway for War and Peace: The Secretory Pathway in Plant–Microbe Interactions. *Molecular Plant* **4**, 581-587.
Wang W-Y, Zhang L, Xing S, Ma Z, Liu J, Gu H, Qin G, Qu L-J. 2012. Arabidopsis AtVPS15 Plays Essential Roles in Pollen Germination Possibly by Interacting with AtVPS34. *Journal of Genetics and Genomics* **39**, 81-92.
Wawrzynska A, Rodibaugh NL, Innes RW. 2010. Synergistic Activation of Defense Responses in Arabidopsis by Simultaneous Loss of the GSL5 Callose Synthase and the EDR1 Protein Kinase. *Molecular Plant-Microbe Interactions* **23**, 578-584.
Wei H, Persson S, Mehta T, Srinivasasainagendra V, Chen L, Page GP, Somerville C, Loraine A. 2006. Transcriptional Coordination of the Metabolic Network in Arabidopsis. *Plant Physiology* **142**, 762-774.
Wei L, Zhang W, Liu Z, Li Y. 2009. AtKinesin-13A is located on Golgi-associated vesicle and involved in vesicle formation/budding in Arabidopsis root-cap peripheral cells. *BMC Plant Biology* **9**, 138.
Wei P-C, Zhang X-Q, Zhao P, Wang X-C. 2011. Regulation of stomatal opening by the guard cell expansin *AtEXPA1*. *Plant Signaling & Behavior* **6**, 740-742.
Whitley P, Hinz S, Doughty J. 2009. Arabidopsis FAB1/PIKfyve Proteins Are Essential for Development of Viable Pollen. *Plant Physiology* **151**, 1812-1822.
Wilson ID, Neill SJ, Hancock JT. 2008. Nitric oxide synthesis and signalling in plants. *Plant, Cell & Environment* **31**, 622-631.
Winter V, Hauser M-T. 2006. Exploring the ESCRTing machinery in eukaryotes. *Trends in Plant Science* **11**, 115-123.
Xiao W, Jang J-C. 2000. F-box proteins in Arabidopsis. *Trends in Plant Science* **5**, 454-457.
Xie C, Zhou X, Deng X, Guo Y. 2010. PKS5, a SNF1-related kinase, interacts with and phosphorylates NPR1, and modulates expression of WRKY38 and WRKY62. *Journal of Genetics and Genomics* **37**, 359-369.
Xu L, Liu F, Lechner E, Genschik P, Crosby WL, Ma H, Peng W, Huang D, Xie D. 2002. The SCFCOI1 Ubiquitin-Ligase Complexes Are Required for Jasmonate Response in Arabidopsis. *The Plant Cell Online* **14**, 1919-1935.
Yang Y, Xu R, Ma C-j, Vlot AC, Klessig DF, Pichersky E. 2008. Inactive Methyl Indole-3-Acetic Acid Ester Can Be Hydrolyzed and Activated by Several Esterases Belonging to the AtMES Esterase Family of Arabidopsis. *Plant Physiology* **147**, 1034-1045.
Yanhui C, Xiaoyuan Y, Kun H, Meihua L, Jigang L, Zhaofeng G, Zhiqiang L, Yunfei Z, Xiaoxiao W, Xiaoming Q, Yunping S, Li Z, Xiaohui D, Jingchu L, Xing-Wang D, Zhangliang C, Hongya G, Li-Jia Q. 2006. The MYB Transcription Factor Superfamily of Arabidopsis: Expression Analysis and Phylogenetic Comparison with the Rice MYB Family. *Plant Molecular Biology* **60**, 107-124.
Yazaki J, Shimatani Z, Hashimoto A, Nagata Y, Fujii F, Kojima K, Suzuki K, Taya T, Tonouchi M, Nelson C, Nakagawa A, Otomo Y, Murakami K, Matsubara K, Kawai J, Carninci P, Hayashizaki Y, Kikuchi S. 2004. Transcriptional profiling of genes responsive to abscisic acid and gibberellin in rice: phenotyping and comparative analysis between rice and Arabidopsis. *Physiological Genomics* **17**, 87-100.
Yonekura-Sakakibara K, Tohge T, Matsuda F, Nakabayashi R, Takayama H, Niida R, Watanabe-Takahashi A, Inoue E, Saito K. 2008. Comprehensive Flavonol Profiling and Transcriptome Coexpression Analysis Leading to Decoding Gene–Metabolite Correlations in Arabidopsis. *The Plant Cell Online* **20**, 2160-2176.
Zhang C, Mallery EL, Schlueter J, Huang S, Fan Y, Brankle S, Staiger CJ, Szymanski DB. 2008. Arabidopsis SCARs Function Interchangeably to Meet Actin-Related Protein 2/3 Activation Thresholds during Morphogenesis. *The Plant Cell Online* **20**, 995-1011.
Zhang J, Li W, Xiang T, Liu Z, Laluk K, Ding X, Zou Y, Gao M, Zhang X, Chen S, Mengiste T, Zhang Y, Zhou J-M. 2010. Receptor-like Cytoplasmic Kinases Integrate Signaling from Multiple Plant Immune Receptors and Are Targeted by a Pseudomonas syringae Effector. *Cell host & microbe* **7**, 290-301.
Zhang JZ, Somerville CR. 1997. Suspensor-derived polyembryony caused by altered expression of valyl-tRNA synthetase in the twn2 mutant of Arabidopsis. *Proceedings of the National Academy of Sciences* **94**, 7349-7355.
Zhang W, Wang C, Qin C, Wood T, Olafsdottir G, Welti R, Wang X. 2003. The Oleate-Stimulated Phospholipase D, PLDδ, and Phosphatidic Acid Decrease H2O2-Induced Cell Death in Arabidopsis. *The Plant Cell Online* **15**, 2285-2295.

Zhang X-Q, Wei P-C, Xiong Y-M, Yang Y, Chen J, Wang X-C. 2011. Overexpression of the Arabidopsis α-expansin gene AtEXPA1accelerates stomatal opening by decreasing the volumetric elastic modulus. *Plant Cell Reports* **30**, 27-36.
Zhang Y, Yang C, Li Y, Zheng N, Chen H, Zhao Q, Gao T, Guo H, Xie Q. 2007. SDIR1 Is a RING Finger E3 Ligase That Positively Regulates Stress-Responsive Abscisic Acid Signaling in Arabidopsis. *The Plant Cell Online* **19**, 1912-1929.
Zheng W, Zhai Q, Sun J, Li C-B, Zhang L, Li H, Zhang X, Li S, Xu Y, Jiang H, Wu X, Li C. 2006. Bestatin, an Inhibitor of Aminopeptidases, Provides a Chemical Genetics Approach to Dissect Jasmonate Signaling in Arabidopsis. *Plant Physiology* **141**, 1400-1413.
Zienkiewicz M, Ferenc A, Wasilewska W, Romanowska E. 2012. High light stimulates Deg1-dependent cleavage of the minor LHCII antenna proteins CP26 and CP29 and the PsbS protein in *Arabidopsis thaliana*. *Planta* **235**, 279-288.
