# Supplementary material for: Partial Activation of SA- and JA-Defensive Pathways in Strawberry upon Colletotrichum acutatum Interaction
Source: Front Plant Sci. 2016 Jul 15;7:1036. doi: 10.3389/fpls.2016.01036 (PMC4945649; doi:10.3389/fpls.2016.01036)
Supplement: Supplementary file 7 [file Image1.PDF]

Title:

Partial activation of SA- and JA-defensive pathways in strawberry upon *Colletotrichum acutatum* interaction

Authors:

Francisco Amil-Ruiz[1], José Garrido-Gala[1], José Gadea[2], Rosario Blanco-Portales[1], Antonio Muñoz-Mérida[3], Oswaldo Trelles[3], Berta de los Santos[4], Francisco T. Arroyo[4], Ana Aguado-Puig[4], Fernando Romero[4], José-Ángel Mercado[5], Fernando Pliego-Alfaro[5], Juan Muñoz-Blanco[1] and José L. Caballero[1*]

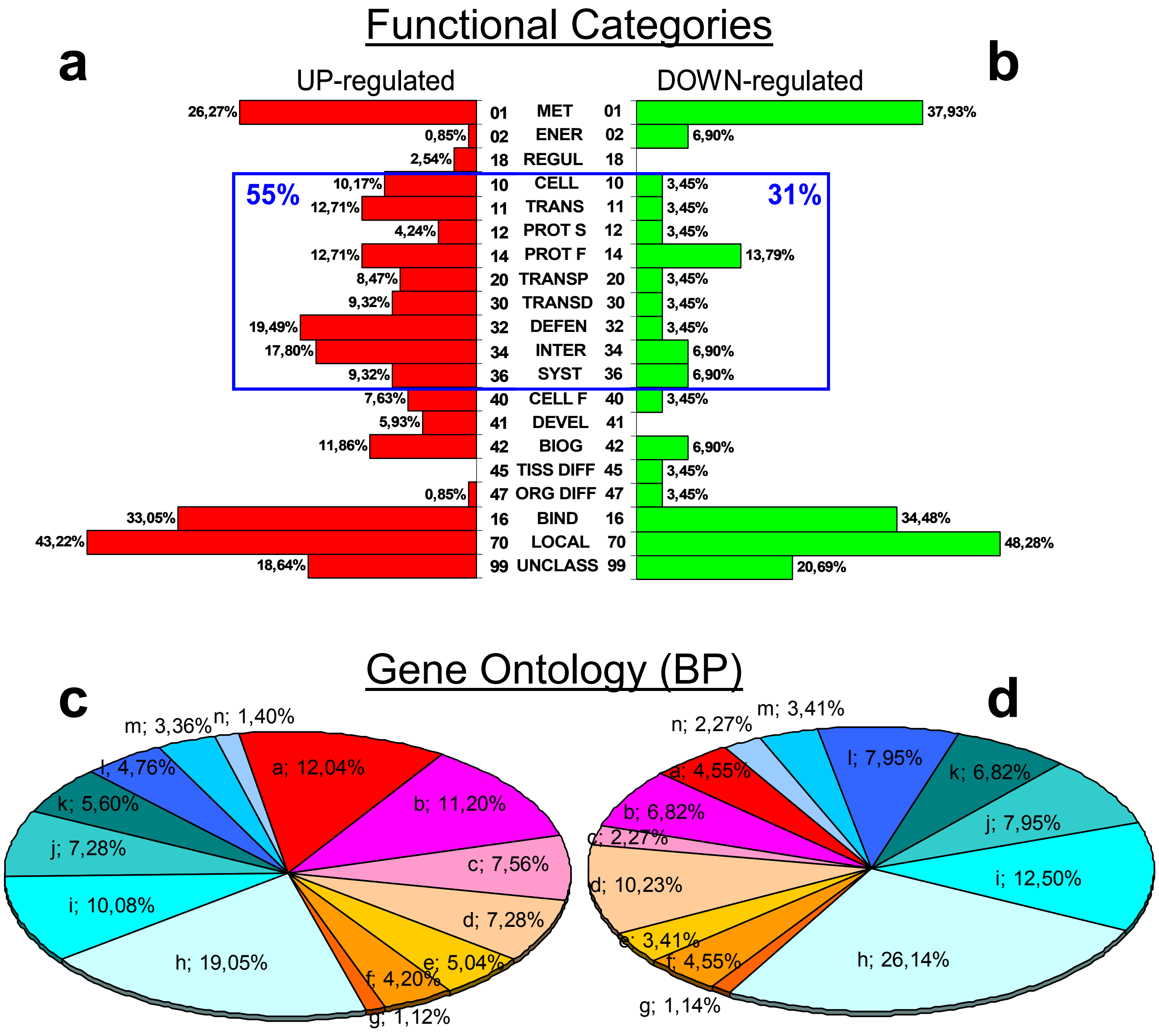

Figure S1. Overview of the microarray results. (a, b) Functional categorization of the differentially expressed genes (a, up-regulated; b, down-regulated, Table S5). Numbers and names have been taken from the functional classification catalogue (FunCat; Ruepp et al., 2004). Percentages represent genes that have been annotated within each function with respect to the total of genes analyzed. According to FunCat, description of the found functional categories is as follow: 01 Metabolism, 02 Energy, 10 Cell Cycle and DNA Processing, 11 Transcription, 12 Protein Synthesis, 14 Protein Fate (Folding, Modification, Destination), 16 Protein With Binding Function or Cofactor Requirement (Structural or Catalytic), 18 Regulation of Metabolism and Protein Function, 20 Cellular Transport, Transport Facilities and Transport Routes, 30 Cellular Communication/Signal Transduction Mechanism, 32 Cell Rescue, Defense and Virulence, 34 Interaction with the Environment, 36 Systemic Interaction with the Environment, 40 Cell Fate, 41 Development (Systemic), 42 Biogenesis of Cellular Components, 45 Tissue Differentiation, 47 Organ Differentiation, 70 Subcellular Localization, 99 Unclassified Proteins. Blue rectangle highline those categories with function in defense response (10, 11, 12, 14, 20, 30, 32, 34, 36). Thus 55% of the up-regulated and 31% of the down-regulated set of genes described in Table I belong to categories related to plant defense and stress response. FunCat categories 32 (19.49%; cell rescue, defense and virulence), 34 (17.80%; interaction with the environment), and 36 (9.32%; systemic interaction with the environment, fungal specific systemic sensing and response) are among the highest represented. Moreover, categories contributing to an integrative plant response to pathogens, such as trafficking facilities and signal transduction, are well represented (8.47% and 9.32%, respectively). Mechanisms such as transcription control (12.71%), protein fate (12.71%), cell cycle and DNA processing (10.17%), and protein synthesis (4.24%), among others, are also represented, and somehow seem to contribute to the global process of strawberry defense. (c, d) Pie chart of gene ontology (GO, Ashburner et al., 2000) at the biological process level, UP and DOWN-regulated genes respectively (Table S6). GO terms shown as follow: a, Response to abiotic or biotic stimulus; b, Response to stress; c, Transport; d, Protein metabolism; e, Signal transduction; f, Transcription, DNA-dependent; g, DNA or RNA metabolism; h, Other metabolic processes; i, Other biological processes; j, Developmental processes; k, Other cellular processes; l, Cell organization and biogenesis; m, Unknown biological processes and n, Electron transport or energy pathways. Terms associated with defense response (a to g) are warm colored. Percentages represent genes that have been associated with each GO term with respect to the total of genes analyzed.
