# Supplementary material for: Partial Activation of SA- and JA-Defensive Pathways in Strawberry upon Colletotrichum acutatum Interaction
Source: Front Plant Sci. 2016 Jul 15;7:1036. doi: 10.3389/fpls.2016.01036 (PMC4945649; doi:10.3389/fpls.2016.01036)
Supplement: Supplementary file 8 [file Image2.PDF]

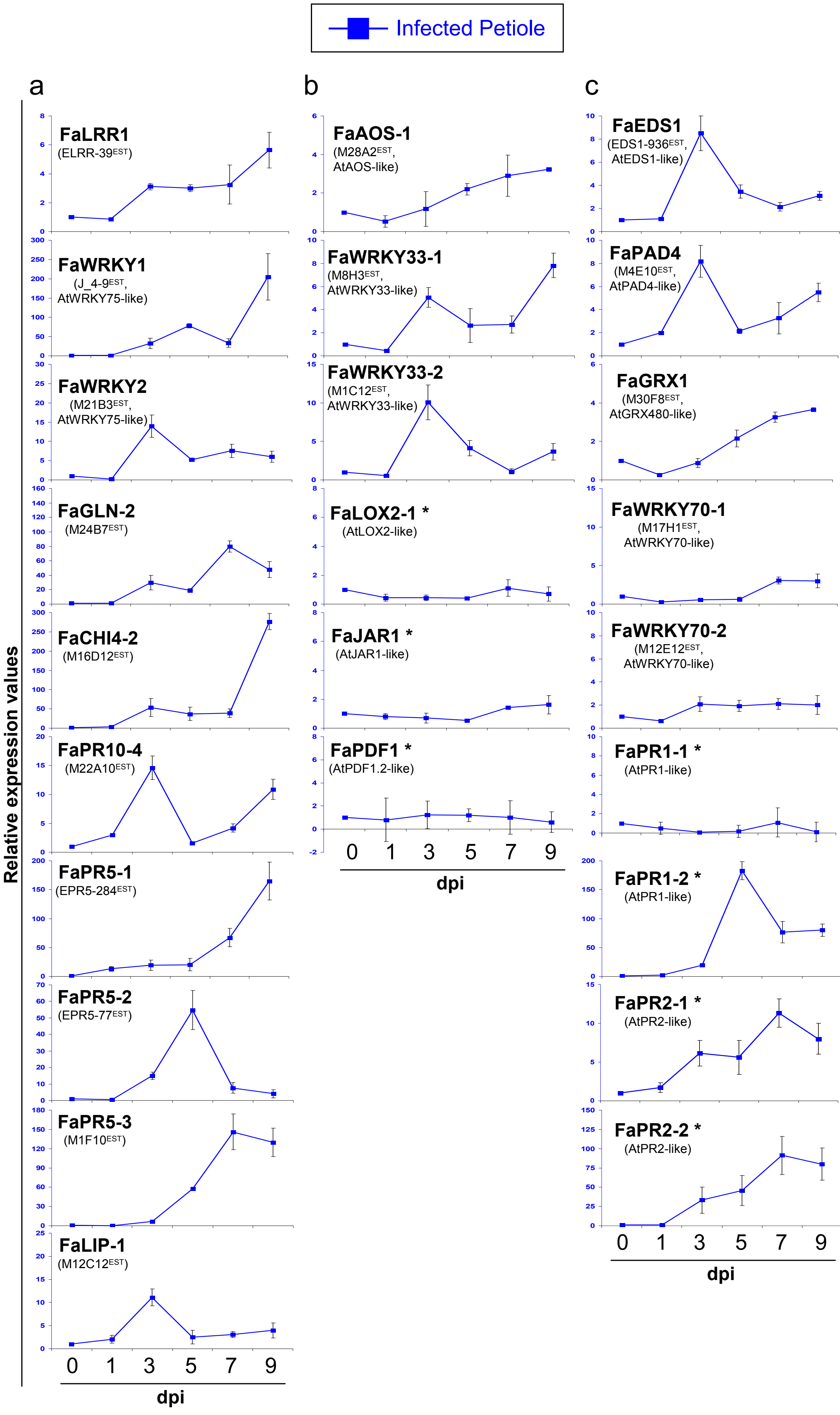


**Figure S2.** Relative expression values by RTqPCR analysis in petiole tissues of upregulated strawberry genes during *C. acutatum* infection. Selected genes correspond to those shown in Figure 2 for crown tissues analysis, (a) relevant strawberry genes in this study, (b) JA-responsive marker genes, and (c) SA-responsive marker genes. . At each time point, every inoculated sample was compared with its corresponding mock treated sample. In the graphics, standard value 1 at T0 was added to better illustrate changes. Asterisk marks genes not present in the Array dataset. Arabidopsis orthologs are *AT5G13080* (*AtWRKY75*), *AT5G42650* (*AtAOS*), *AT2G38470* (*AtWRKY33*), *AT3G45140* (*AtLOX2*), *AT2G46370* (*AtJAR1*), *AT5G44420* (*AtPDF1.2*), *AT3G48090* (*AtEDS1*), *AT3G52430* (*AtPAD4*), *AT1G28480* (*AtGRX480*), *AT3G56400* (*AtWRKY70*), *AT2G14610* (*AtPR1*), *AT3G57260* (*AtPR2*).
